# Perspectives on the Impact of Varicella Immunization on Herpes Zoster. A Model-Based Evaluation from Three European Countries

**DOI:** 10.1371/journal.pone.0060732

**Published:** 2013-04-17

**Authors:** Piero Poletti, Alessia Melegaro, Marco Ajelli, Emanuele del Fava, Giorgio Guzzetta, Luca Faustini, Giampaolo Scalia Tomba, Pierluigi Lopalco, Caterina Rizzo, Stefano Merler, Piero Manfredi

**Affiliations:** 1 Center for Information Technology, Bruno Kessler Foundation, Trento, Italy; 2 DONDENA Centre for Research on Social Dynamics, Bocconi University, Milan, Italy; 3 Center for Statistics, University of Hasselt, Diepenbeek, Belgium; 4 ISTAT Regional Office for Tuscany, Firenze, Italy; 5 Department of Mathematics, University of Rome-Tor Vergata, Italy; 6 European Centre for Disease Prevention and Control, Stockholm, Sweden; 7 National Center for Epidemiology Surveillance and Health Promotion, Istituto Superiore di Sanità, Rome, Italy; 8 Department of Statistics and Mathematics Applied to Economics, University of Pisa, Pisa, Italy; Fundacion Huesped, Argentina

## Abstract

The introduction of mass vaccination against Varicella-Zoster-Virus (VZV) is being delayed in many European countries because of, among other factors, the possibility of a large increase in Herpes Zoster (HZ) incidence in the first decades after the initiation of vaccination, due to the expected decline of the boosting of Cell Mediated Immunity caused by the reduced varicella circulation. A multi-country model of VZV transmission and reactivation, is used to evaluate the possible impact of varicella vaccination on HZ epidemiology in Italy, Finland and the UK. Despite the large uncertainty surrounding HZ and vaccine-related parameters, surprisingly robust medium-term predictions are provided, indicating that an increase in HZ incidence is likely to occur in countries where the incidence rate is lower in absence of immunization, possibly due to a higher force of boosting (e.g. Finland), whereas increases in HZ incidence might be minor where the force of boosting is milder (e.g. the UK). Moreover, a *convergence* of HZ post vaccination incidence levels in the examined countries is predicted despite different initial degrees of success of immunization policies. Unlike previous model-based evaluations, our investigation shows that after varicella immunization an increase of HZ incidence is not a certain fact, rather depends on the presence or absence of factors promoting a strong boosting intensity and which might or not be heavily affected by changes in varicella circulation due to mass immunization. These findings might explain the opposed empirical evidences observed about the increases of HZ in sites where mass varicella vaccination is ongoing.

## Introduction

Varicella, commonly referred to as chickenpox, is a highly transmissible infection caused by Varicella-Zoster-Virus (VZV), a DNA virus of the Herpes group, transmitted by direct contact with infective individuals. In Europe, 90% of children get infected with VZV before 12 years of age and around 95% of adults are immune to VZV [1]. After recovery from varicella infection, the VZV virus remains latent in the dorsal root ganglia where it can reactivate at later ages, causing Herpes Zoster (HZ), an inflammatory skin disease (also known as shingles), which might cause significant morbidity, including the long and painful post-herpetic neuralgia [2], as well as high costs to public health payers and societies [3], [4].

The mechanisms underlying the development of HZ and, more generally, the relation between HZ and varicella infection/exposure are still poorly understood. The prevailing view, dating back to Hope-Simpson seminal paper, is that after temporary immunity following varicella infection, VZV reactivation may occur due to the decline of Cell Mediated Immunity (CMI) [5]–[7], e.g. as a consequence of ageing [8] or other immune-suppressing processes [9] and lead to the development of HZ. Still in the same paper, Hope-Simpson introduced the hypothesis of *exogenous boosting*, i.e. that re-exposure to VZV may be protective against HZ through boosting of CMI. Although some controversy on this hypothesis has been raised [10], to the best of our knowledge alternative hypotheses, e.g. that boosting might follows from immunological phenomena within the individual, did not receive empirical support. On the opposite, substantial evidence has accumulated in favour of “exogenous boosting hypothesis” from a variety of studies ranging from field studies [11], to model-based evidence [12], to Immunological and epidemiological studies [1], [13].

A live attenuated varicella virus vaccine (Oka strain) has been available since the 70 s and in 1995 a universal childhood vaccination program against varicella infection was introduced in the US. However, although the vaccine has been shown to be safe [14], [15] and largely effective against varicella [15]–[20], the introduction of mass VZV immunization programs in Europe is stalled, with only a few countries (Germany, Greece and Luxembourg) and regions (e.g., Sicily, Tuscany and Veneto in Italy and the Autonomous Community of Madrid in Spain) currently vaccinating routinely [21]. Contrary to expectations, even the availability of a quadrivalent measles, mumps, rubella, and varicella vaccine (MMRV) did not facilitate the decision making process for introducing universal varicella vaccination in many European countries [22].

The reasons for different current policies are doubts about the possible consequences of varicella vaccination on the epidemiology of chickenpox (i.e. shift in the age at infection and varicella among vaccinated children) but also, to a large extent, about a possible increase of HZ incidence due to the reduction of immunological boosting caused by the varicella vaccination.

The latter effect has been shown in mathematical models, incorporating the assumption of CMI boosting effect, predicting an increase of HZ incidence in the first 3–5 decades following the introduction of vaccination [12], [23]–[28]. The argument is that the decline in VZV circulation following mass immunization will dramatically decrease the number of episodes of CMI boosting, thereby increasing the risk of developing HZ. Unfortunately, the empirical evidence from sites where varicella mass vaccination programs are ongoing is still controversial, with some studies supporting the hypothesis of HZ increase and others not [2], [15], [29], [30].

In this paper, we aim to contribute to the debate on the introduction of varicella vaccine in Europe by using an innovative framework for robustly assessing the impact of VZV mass vaccination on HZ epidemiology, using data from three European countries with remarkable differences in the epidemiology of both varicella and HZ: Italy, Finland and the UK.

Compared to past VZV modeling efforts [12], [23]–[28], the framework developed here is innovative in three main respects. First, we address (to the best of our knowledge, for the first time) the challenge of appropriately handling the *structural* uncertainty on HZ parameters, related to the lack of data on and knowledge about the process through which individuals acquire HZ susceptibility. Second, in order to further reduce the uncertainty of HZ parameter estimates, we fitted the model simultaneously to HZ data from the three selected countries, with the purpose of better estimating those parameters that, having a biological basis, shouldn’t show a significant inter-country variation. Third, no arbitrary assumptions are made about vaccine-related parameters which are incorporated in the model with uncertainty distributions with wide ranges.

Despite the uncertainty thus incorporated, which results in a wide uncertainty about the impact of the vaccine on varicella epidemiology, the model provides surprisingly robust predictions of the impact of VZV vaccination on HZ epidemiology in the different countries. An increase of HZ incidence is not expected to occur in all countries but rather seems to depend on the presence (Finland) or absence (Italy and the UK) of factors which promote a strong intensity of boosting and thus may (or may not) be heavily affected by changes in the circulation of the virus due to vaccination campaigns.

Overall, we feel that the different impacts of vaccination predicted in the three countries considered, which stem from differences in VZV and HZ epidemiology between countries, might be fairly representative of Europe as a whole. Therefore, although modeling VZV and HZ is a complex task due to the poor understanding of basic processes, we believe that the present results may contribute to better predictions of the effects of introducing VZV mass vaccination and thus assist current and future decision making.

## Methods

### Data

Pre-vaccination age-specific VZV seroprevalence data for Finland, Italy and the UK were made available from the European Seroepidemiological Network 2 [1], which is the largest available population-based study on VZV seroprevalence in Europe. The method of sera collection adopted (residual sera) is the same in the three countries considered in this paper. The results from the assays adopted in the ESEN2 study are standardized which make international comparisons possible. The total sample sizes were in the range (about 2000) considered optimal for these types of studies, and all studies were designed to be representative by age. These data describe the natural history of infection and are critical for estimating the force of infection (FOI) of varicella. Age-specific HZ case notification data were obtained from published studies for Finland [28], Italy [3] and United Kingdom [23]. Country-specific routine socio-demographic data for the three different countries were obtained from the Eurostat databases [31], for estimation of contact distributions.

### The Mathematical Model for VZV Transmission and HZ Incidence

**Figure 1:**
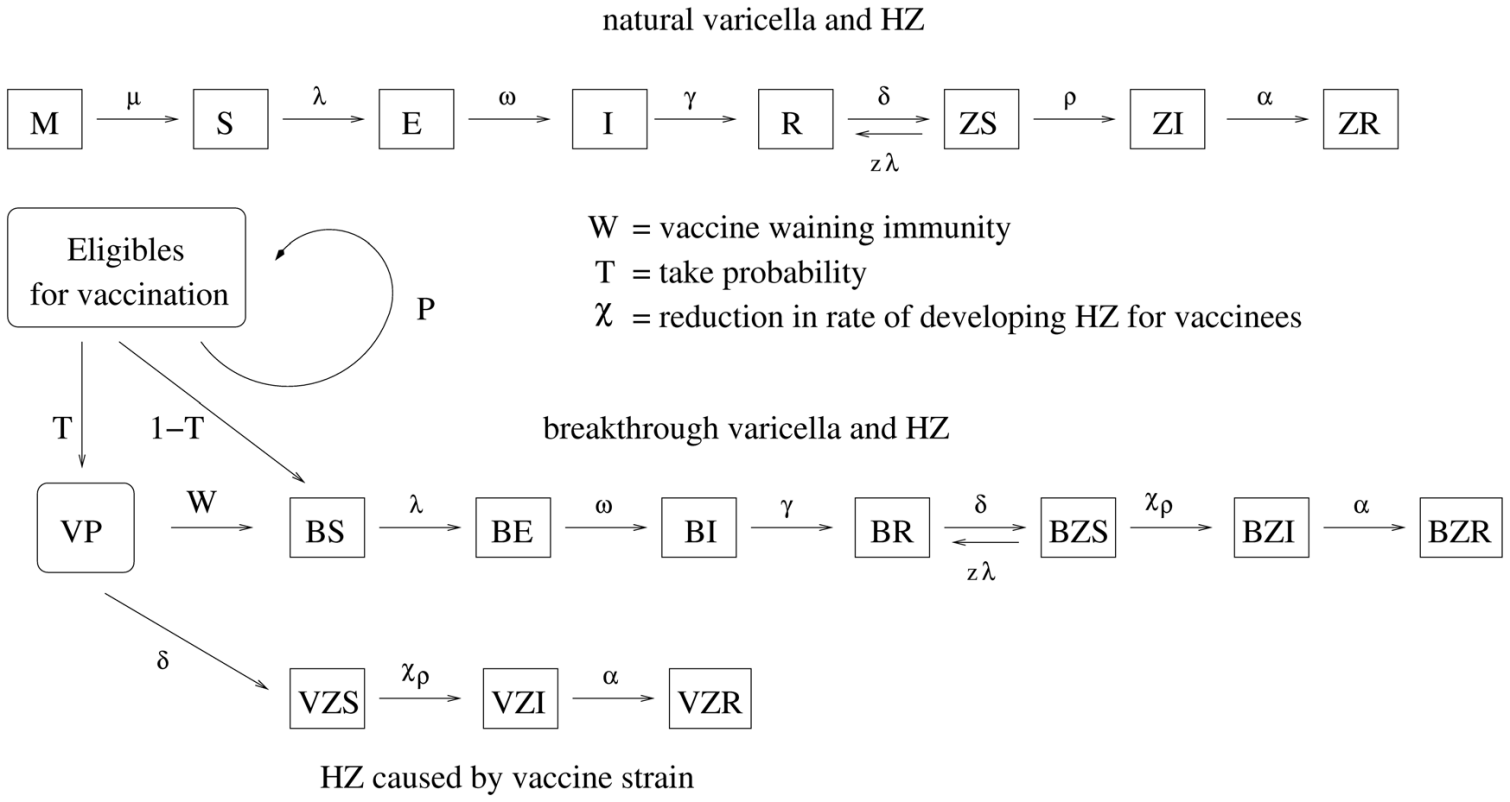
Schematic representation of the model. Natural varicella and HZ. M represents individuals protected by maternal antibodies; VS, VE, VI represent varicella susceptible, latent and infective; VR represents individuals recovered from varicella and temporally protected by CMI against HZ; ZS, ZI, ZR represent individuals susceptible, infected and permanently immune to HZ. **Vaccine protection, varicella and HZ among vaccines.** VP represents vaccine protected individuals. VBS, VBE and VBI represent vaccinated individuals susceptible, latent and infective for breakthrough varicella; VBR represents individuals recovered from breakthrough varicella and temporally protected by CMI against (breakthrough) HZ. ZBS, ZBI, ZBR represent individuals susceptible, infected and permanently immune to HZ that experienced varicella breakthrough after vaccination. ZVS, ZVI, ZVR represent individuals susceptible, infected and permanently immune to HZ for the vaccine strain. Details in Text S1.

The mathematical model for VZV transmission dynamics and HZ development extends earlier compartmental models [12], [25], [27], [32] by incorporating different country-specific levels of boosting (flow diagram in Fig. 1). Full technical details are given in Text S1. Briefly, newborn individuals are assumed to be protected by maternal antibodies for six months on average [33], after which they become susceptible to varicella and are exposed to an age-specific FOI 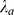. In the absence of vaccination, the FOI is defined as follows: 
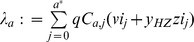
 where 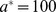 as we consider a population subdivided in 100 1- year age classes, *q* is an age-independent transmission coefficient, according to the so-called *social contact hypothesis* [34]; 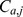 is an age-specific contact matrix whose entries describe the average numbers of different persons in age group j encountered by an individual belonging to age group a per unit of time (so that the product 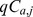 defines the number of adequate contacts for having infection transmission); 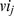 and 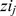 are, respectively, the fractions of varicella and HZ cases of age j in the population and 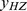 is the relative VZV infectiousness associated with HZ; latency and infectious periods are assumed to last, respectively, 14 and 7 days on average [33], [35], [36]. After recovery, individuals become permanently immune to varicella. VZV immune individuals become susceptible to HZ due to the decline in CMI, which occurs at constant rate 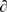. HZ susceptibles have two possibilities, either boosting their CMI by exposure to infectious individuals, thus reacquiring the protection against the development of HZ, or progressing to HZ disease, at a rate 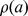 which is higher in children and the elderly and lower in adults [25]–[27]. HZ cases contribute to the varicella force of infection 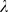 for 7 days on average [36]; afterwards, they become permanently immune to HZ disease. The CMI of HZ susceptible individuals is boosted according to a *force of boosting* (FOB) 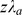, which is assumed to be proportional to the varicella FOI.

The incomplete protection supplied by the licensed varicella vaccine against varicella and HZ [15], [17], [18], [20], [37]–[43] makes the post-vaccination model articulated in several cases. A proportion P of vaccinated individuals suffer an initial vaccine failure and remain susceptible, while the complementary fraction of vaccinated individuals (1-P) have a fixed *take* probability T to acquire temporary protection against VZV infection and a lifelong partial protection against HZ. For the sake of simplicity in model simulation, we assume that vaccine coverage accounts also for primary failure. This means that e.g. when we consider a coverage of 90%, we assume that a possibly higher proportion of individuals is vaccinated, but only 90% are vaccinated without experiencing the primary vaccine failure. As a consequence, when the coverage is set to 100%, primary failure probability is assumed to be zero. Vaccine protected individuals lose immunity against varicella at a waning rate W.

Vaccinated individuals who suffer a secondary failure (a fraction (1–T)(1-P)) and vaccine protected individuals whose immunity against varicella has waned are exposed to the natural FOI, but are assumed to acquire a milder infection commonly referred as *breakthrough varicella* which will be less infectious than natural varicella (i.e., varicella occurring among unvaccinated individuals). Vaccinees can develop HZ after breakthrough varicella or directly from the vaccine strain [6]. In the latter case, HZ is caused by vaccine virus instead of wild type. The mechanism of varicella and HZ development among vaccinees is the same as the one described for individuals who experience natural varicella infection. However the rate at which vaccinated HZ susceptibles develop HZ is lower [44] (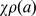
instead of 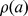 where 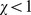). The post-vaccination varicella FOI becomes: 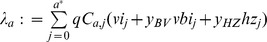 where 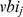 is the fraction of breakthrough varicella cases of age j and 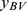 is their relative VZV infectiousness; 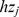 is the fraction of HZ cases of age j. A full listing of model parameters and literature sources is given in Table 1.

**Table 1:** Parameters employed.

Parameter	Interpretation	Value
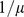	Average duration of the maternal antibodies protection	6 months [23]
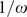	Average duration of the latency period	14 days [27]
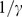	Average duration of the varicella infectivity period	7 days [27]
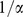	Average duration of the HZ infectivity period	7 days [27]
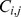	Age specific contact matrices (different for each countries)	based on census data [31]
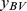	Relative VZV infectiousness of breakthrough varicella cases	0.5 [43]
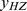	Relative VZV infectiousness of HZ cases	0.05 [23]
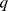	Adjusting factor for contacts relevant for VZV transmission	fitted against the VZV seroprevalence
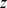	Adjusting factor for contacts relevant for the boosting of CMI (boosting component) accounting for the uncertainty of FOI in adults and the elderly	fitted against HZ incidence
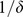	Average duration of the CMI	fitted against HZ incidence
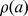	Age dependent VZV reactivation rate 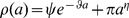	fitted against HZ incidence
*T*	Vaccine take (probability)	unif. sampled from (0.8,1) [15], [17], [37], [39], [42]
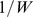	Average duration of vaccine waning immunity	unif. sampled from (1,200) years [52]
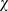	Reduction factor of the risk of developing HZ for vaccinees	unif. sampled from (1/12,1/4) [44]

Model parameters, description and values considered for simulations.

### Model Parameterization

Model parameterization involved several steps. First, age-specific contact matrices for each country considered were taken from [45] **.** These contact matrices were computed by an approach similar to the one introduced in [46] but further developed to derive social contact structures for several European countries. Briefly, a synthetic population of agents, each one corresponding to an individual in the real population, is generated by using highly detailed routine socio-demographic data [31], such as household distributions by type, size and age of members, school sizes for the various school levels, workplace sizes and employment rates by age, and the age distribution of the general population. From this synthetic population, age-specific contact matrices for each country considered were computed. These synthetic matrices are possibly more harmonized compared to Polymod matrices and less prone to observational biases. Nonetheless, they share several common features with the Polymod matrices, e.g. strong assortativeness and the presence of similar secondary diagonal contact pattern, and as shown in [45], most statistical variation between Polymod-type and synthetic matrices can be captured by a single scale factor.

**Figure 2:**
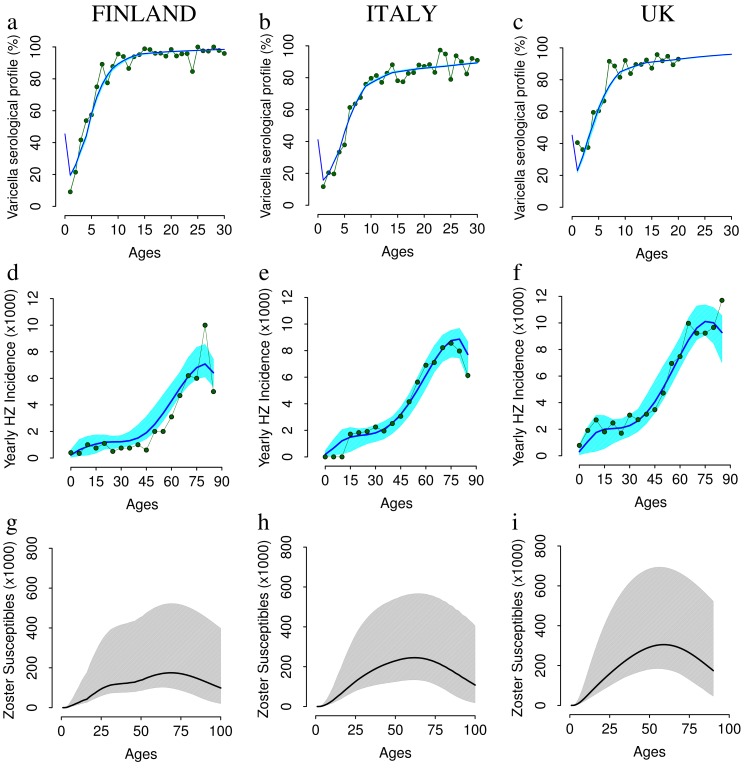
VZV seroprevalence, HZ incidence and boosting incidence in Finland, Italy and the UK. Top Row. VZV seroprevalence by age as observed in [1] (in green) and as predicted by the model (average in blue, 95% CI in cyan) for Finland (a), Italy (b), UK (c). Mid Row. Yearly HZ incidence by age(cases per 1,000 individuals) as observed in [3], [23], [28] (in green) and as predicted by the model (average in blue, 95% CI in cyan) for Finland (d), Italy (e), UK (f); Bottom row. Predicted HZ susceptibility age profile for Finland (g), Italy (h), UK (i). Results are based on 1,000 model realizations.

Second, age-specific FOI estimates for varicella in the three selected countries were estimated by fitting an age-structured SIR model at endemic equilibrium to age-specific varicella serological data (Fig. 2abc) and estimating the three country-specific transmission coefficients q (as in [34], [46]–[49]) which, together with the synthetic contact matrices, maximized the likelihood of the varicella seroprofiles observed in each country by assuming a negligible contribution of HZ to the pre-vaccination varicella FOI (details in Text S1).Third, parameters describing progression to HZ disease (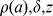) were estimated by fitting the whole pre-vaccination model to HZ age-specific incidence data conditionally on the estimates found for varicella transmission and by minimizing the mean squared error between observed and predicted HZ incidence (Fig. 2def) in the different countries.

Considering the difficulties related to HZ susceptibility status being non observable, we favored a novel approach to parameter fitting which we call *simultaneous*, whereby HZ parameters were estimated using age-specific zoster incidence data from all the three countries together assuming that parameters describing strictly biological processes (such as the average duration of CMI and the VZV reactivation rates) were the same in all countries, while parameters reflecting social interactions might be different. This approach was preferred to the traditional one, based on separate *parallel* fitting for each country, as the latter might yield inconsistent inter-country estimates of *biologically* based HZ parameters (see further details in Text S1). On the other hand, we allowed the scaling factor z of CMI boosting to be country-specific. Indeed, this parameter is not a purely biological parameter but may depend also on social factors, e.g. contacts of the elderly, and as such may account for the large uncertainty in the FOI, and therefore in the FOB, at higher ages.

Unlike previous modeling work [12], [23]–[26], where some vaccine related parameters were estimated from vaccine trials data using simple dynamic models and making a-priori assumptions on the duration of CMI, we decided to rely on literature estimates. Our simulations include the reduction factor 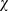 in the reactivation rate for vaccinated individuals documented in [44], where it is shown that individuals with a history of varicella vaccination have a 4 to 12 times lower risk of developing HZ compared to individuals with a history of natural infection (further uncertainty on 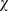 is investigated in Text S1). However, we preferred to explore a wide range of possible values for the waning rate W and the take probability T in order to account for the large uncertainty characterizing these parameters [7], [50]–[52].

### Immunization Scenarios

The impact of VZV vaccination on HZ incidence is investigated under different scenarios of coverage and schedule. The program consisting in one single dose provided to 1 year old children is analyzed by assuming coverage levels ranging from 70% to 100%. Moreover, a two-dose program has been suggested to be more effective and appropriate [15], [17], [18], [20] and the Advisory Committee on Immunization Practices (ACIP) in the US modified its recommendations to a routine 2-dose program in 2006 [18]. As a matter of fact, given the reported number of breakthrough cases in countries where vaccination is in place, a two-dose program seems to be unavoidable [15], [38], [39]. Thus, a two dose program was also considered with the first dose administered to 1 year old children and the second one to 5 years old children. Coverage of 90% and 80% have been assumed for the first and the second dose respectively. To sum up, we consider the following immunization scenarios: a single dose program, with the vaccine administered to 1 year old children with 70%, 80%, 90% and 100% coverage, and the two-dose program described above.

### Uncertainty Evaluation

Uncertainty in model outputs depends on the uncertainty in three classes of input parameters, i.e. those related to (a) varicella transmission, (b) HZ development, (c) vaccine-related parameters. Specifically, for vaccine-related parameters, uniform distributions were assumed over the ranges reported in Table 1. For the *structural* uncertainty characterizing all HZ parameters, which implies that there are many parameter configuration which *best fit* HZ incidence data, we decided to take into account all these *best fitting* configurations. Bootstrap techniques were used to evaluate uncertainties about VZV transmission (i.e. q) and parameters related to HZ development (see Text S1). Moreover, sampling from the various uncertainty distributions of parameters was performed by Latin-Hypercube-Sampling (LHS) method [53].

## Results

### The Pre-vaccination Epidemiology of Varicella and HZ in Italy, Finland and the UK

The observed varicella seroprofiles for Italy, Finland and the UK are remarkably different (see Fig. 2a,b,c). In Finland, the fraction of population aged 10–19 immune to varicella is, on average, 6% larger than in the UK and 13% larger than in Italy, indicating a relatively higher FOI in Finland. In agreement with this and based on contact matrices described in the methods, we obtained values of the basic reproductive number 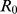 (i.e. the average number of secondary infections resulting from a single infectious individual in a fully susceptible population [54]) also remarkably different across countries: 3.36 (95% CI 3.26,3.48) in Italy, 6.86 (95% CI 6.40,7.17) in Finland and 4.67 (95% CI 4.51,4.90) in the UK (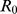
distributions can be found in Text S1). Values of R0 obtained here are compliant with previous estimates provided by using different contact matrices [49]. However, it is worth noting that the obtained ranking in the transmissibility potential among countries, cannot be extended in general for other childhood disease (e.g. pertussis [55]), given the different natural and epidemiological history of each single disease.

Age-specific HZ incidences in the three countries show similar profiles (low and constant rates among young individuals, then a rapid increase in adults, though the pattern is less clear in the very old, e.g. in Italy there seems to be a decline), but also remarkable differences in scale, with the UK rates being twice as high as the ones from Finland (see Fig. 2d,e,f).

By ascribing these differences to varicella transmissibility and HZ susceptibility, while keeping biological HZ related parameters constant across countries, we were able to well reproduce both serological varicella data (Fig. 2abc) as well as the observed HZ incidence (Fig. 2d,e,f).

More specifically, given that CMI duration and the reactivation rate were simultaneously fitted on the three country-specific datasets, the major between-country difference was the predicted role of boosting, which resulted stronger in Finland compared to Italy and the UK. This role is well illustrated (Fig. 2g,h,i) by the differences in the predicted age profile of HZ susceptibility among individuals, which is remarkably lower in Finland compared to Italy and the UK. This result is consistent with the observed scale of HZ incidence, which is about twice as large in the UK as in Finland (Fig. 2d,e,f). The somewhat counterintuitive interpretation is that though Finland suffers a higher varicella FOI, i.e. more (and occurring earlier) varicella infection, thereby creating more space for HZ, this higher transmissibility also yields a stronger boosting effect, and therefore ultimately a fewer overall number of HZ cases in the population. Given the remarkable inter-country epidemiological differences in terms of both age specific HZ incidence and VZV transmissibility potential, the impact of varicella vaccination on HZ epidemiology is expected to be strongly country-specific.

The predicted age distribution of boosting episodes is similar among countries (see Text S1) and it is characterized by two peaks, the first one at ages 5–10, the second one at ages 20–50, probably determined by contacts of infected children with siblings and/or schoolmates in the first case and with parents in the second. Moreover, the reactivation rate is predicted to decline from birth to about age 30 and starting to increase thereafter (see Text S1). This is consistent with the idea that immune competence is not completely developed in young children and that it decreases with age. The average duration of CMI is estimated to be in the range of 60–100 years, which is surprisingly different from the one estimated in previous studies (i.e. 20 years in Brisson et al. [12] and following papers). Though seemingly different from previous estimates, these values are fully compatible with the obtained 30% HZ lifetime risk [12]. For example, for 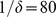 years, given the exponential decline in CMI levels after varicella immunity, 39% of those who had varicella are - in absence of boosting - already susceptible to zoster 40 years after varicella infection (i.e. essentially at ages 40–49). Even if boosting is considered, the risk of HZ is higher than the FOB at high ages, which implies that a substantial proportion of adult HZ susceptible will get HZ prior to being boosted.

We investigated carefully the case of perfect boosting (z = 1 for all countries i.e., FOB equal to country-specific FOIs) and our conclusions are that the model fails to reproduce the country specific HZ incidences unless we relax our principal hypothesis that the duration of CMI and the reactivation rate are the same in all countries. Indeed, following the traditional approach, based on separate *parallel* fitting, the model is able to well reproduce the HZ incidences, though yielding strongly inconsistent inter-country estimates of CMI duration i.e., 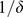 in the range of 20–80 for the UK and in the range of 120–160 for Finland (see Text S1 for more details).Therefore, all results presented hereafter are based on the *simultaneous* fitting approach whereas the parallel fit is considered only for sensitivity analyses.

### The Impact of Vaccination on Varicella

**Figure 3:**
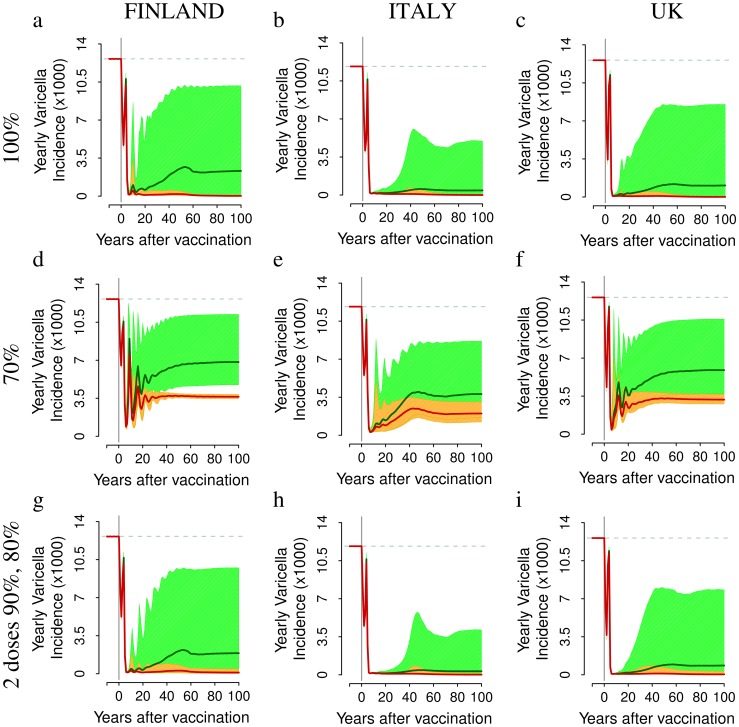
The impact of VZV vaccination on varicella incidence. Top row. Yearly incidence of varicella (average in dark green, 95% CI in light green) and of natural varicella (average in red, 95% CI in orange) per 1,000 individuals as predicted by simulating a single vaccine dose administered to 1 year-old infants with 100% coverage in Finland (a) in Italy (b) and in the UK (c). Mid row. As the top row but obtained by considering 70% coverage in Finland (d) in Italy (e) and in the UK (f). Bottom row. As the top row but for the two-dose scenario, which assumes the administration of a first dose to 1 year-old individuals (90% coverage) and a second dose to 5 years-old individuals (80% coverage) in Finland (g) in Italy (h) and in the UK (i). Results are based on 1,000 model realizations.

Under all programs considered, a remarkable decrease in varicella incidence after vaccination is predicted, on average, in the medium and long term in all countries considered (main scenarios reported in Fig. 3, alternative scenarios reported in Text S1). However, a large uncertainty surrounds the average predictions, which is essentially the consequence of the uncertainty surrounding vaccine related parameters. Cases of natural varicella are predicted to decrease as the mass immunization program progresses. However, in general, even with 100% coverage, varicella cannot be eliminated with a single dose administered to 1 year-old infants (See Fig. 3a,b,c), unless vaccine protection wanes remarkably slowly and the probability of vaccine failure is sufficiently small. As expected, vaccination is predicted to be less effective in reducing varicella incidence in countries where the VZV transmissibility potential (R0) is large, as in Finland. As a rule, there is an initial phase after the introduction of the vaccine where virus circulation is sustained by older unvaccinated individuals. However, due to vaccine failure and waning immunity, in less than 10 years (on average) breakthrough varicella becomes the main source of VZV transmission. This is supported by the analysis of empirical data [19] and also found in other modeling studies [12], [23]–[27]. Specifically, when 100% coverage is considered, most varicella infections in the medium and long term are breakthrough cases while for lower coverage levels, the fraction of natural varicella infections remains large. Indeed, in the 70% coverage scenario (Fig. 3def), the long term (after about 50 years) fraction of total predicted cases due to natural varicella is 53.3% (95% CI 34.4,73.1) for Finland, 52.9% (95% CI 35.5,75.2) in Italy, and 54.0% (95% CI 34.8,74.9) in the UK. Under the two-dose program (90% +80% coverages), the predicted impact of vaccination on varicella in the three different countries is similar to the one predicted for a single dose with 100% coverage (see Fig. 3a,b,c and Fig. 3g,h,i).

Model simulations suggest the possibility that incidence of breakthrough varicella might be very relevant in the long term. For instance, a scenario where breakthrough varicella may be as high as 10 per 1000 individuals per year in Finland i.e., very close to the pre-vaccination varicella incidence (about 12 per 1000 individuals per year) cannot be excluded.

**Figure 4:**
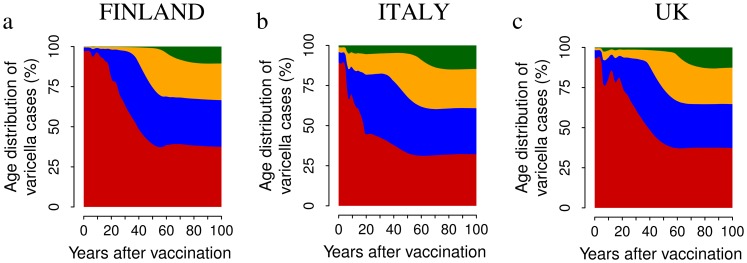
The impact of VZV vaccination on the age distribution of varicella cases. Average yearly distribution of varicella cases among individuals aged 0–20 (red), 21–40 (blue), 41–60 (orange) and 61+ (green) as predicted by simulating a single vaccine dose administered to 1 year-old infants with 100% coverage in Finland (a) Italy (b) and the UK (c). Results are based on 1,000 model realizations.

Moreover, model predictions show that the age at varicella infection increases in all considered scenarios (See Fig. 4 and Text S1). Specifically, in Finland, before the introduction of vaccination, less than 5% of varicella cases are predicted to occur among individuals older than 20. If a single-dose program with 100% coverage is considered, after 20 years of vaccination, the proportion of cases occurring in Finland among individuals older than 20 is predicted to increase to 20% and to more than 50% after 50 years of VZV vaccination. Similar results have been obtained for Italy and the UK. The effect is milder when considering lower vaccine uptake. However, in the medium-long term, the fraction of cases occurring in individuals older than 20 is predicted to increase from 5% to at least 25% for all coverages and countries (details in the Text S1).

To sum up, in the long term we expect essentially all breakthrough infections mostly occurring in adults and the elderly. Finally, as expected, VZV mass vaccination results in a dramatic decline of yearly boosting incidence (details in Text S1), namely 78% in Finland, 97% in Italy and 94% in UK when 100% coverage is considered.

### The Impact of Vaccination on HZ Incidence

There is a striking difference between model predictions on HZ compared to varicella: short and medium-term predictions on HZ are surprisingly robust, unlike the ones on varicella, because these predictions mainly concern individuals who acquired varicella before the start of vaccination. Consequently these predictions are not affected by the large uncertainty characterizing vaccine-related parameters (i.e., the corresponding prediction intervals are surprisingly narrow).

**Figure 5:**
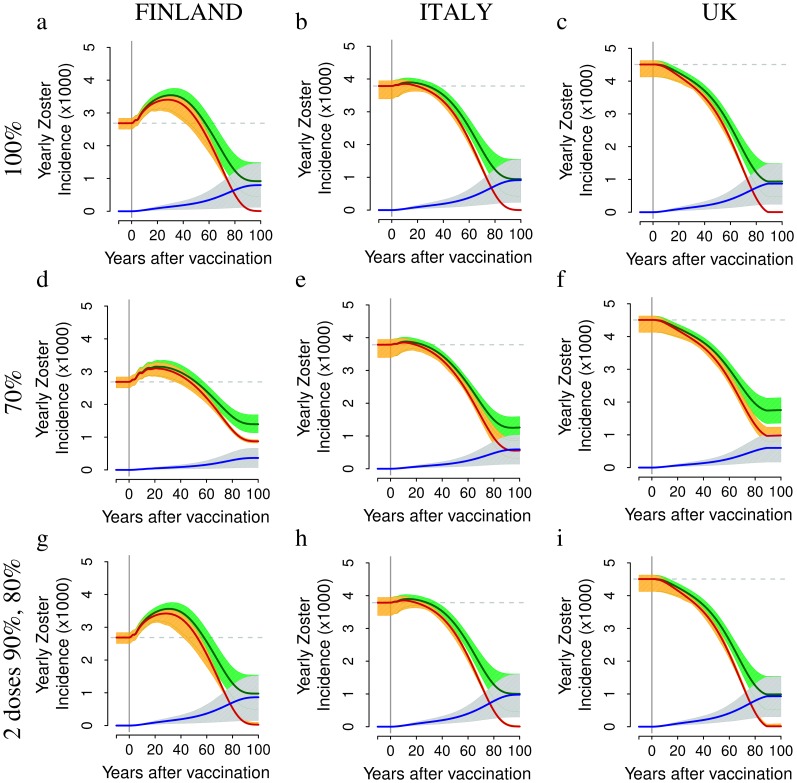
The impact of different vaccination schedules and coverages on HZ incidence. Top row. Yearly incidence of HZ (average in dark green, 95% CI in light green), of natural HZ (i.e., HZ cases occurring among unvaccinated individuals that have experienced natural varicella (average in red, 95% CI in orange) and of HZ caused by the vaccine strain (average in blue, 95% CI in light blue) per 1,000 individuals as predicted by simulating a single vaccine dose administered to 1 year-old infants with 100% coverage in Finland (a) in Italy (b) and in the UK (c). Mid row. As the top row but obtained by considering 70% coverage in Finland (d) in Italy (e) and in the UK (f). Bottom row. As the top row but for the two-dose scenario, which assumes the administration of a first dose to 1 year-old individuals (90% coverage) and a second dose to 5 years-old individuals (80% coverage) in Finland (g) in Italy (h) and in the UK (i). Results are based on 1,000 model realizations.

The predicted impact of VZV vaccination on HZ incidence is strongly country-specific (main scenarios reported in Fig.5, alternative scenarios reported in the Text S1). In Finland, HZ incidence is predicted to increase by 17–32% (depending on coverage and number of doses) for about 40–60 year after the start of immunization (Fig5 a,d,g), whereas in Italy, where the observed HZ incidence in absence of immunization is larger and the force of infection of varicella is lower, the increase is much smaller (+2.5–3%, Fig 5 b,e,h). Finally, in the UK (Fig. 5 c,f,i), HZ results remarkably mitigated by vaccination. More specifically, in Finland HZ incidence is expected to increase from 2.69 (95% CI 2.50,2.84) to 3.54 (95% CI 3.13,3.75) per 1000 individuals per year at 30 years post vaccination, when the 1-dose program with 100% coverage is considered (Fig. 5a) and up to 3.15–3.45 per 1000 when lower coverages (70%) are considered (Fig. 5d). Similarly, in the two-doses program, the HZ incidence is expected to increase to 3.53 (95% CI 3.14,3.74) per 1000 (Fig. 5g). In Italy (Fig. 5beh), HZ incidence is instead predicted to increase only slightly from about 3.79 to about 3.88–3.90 per 1000 individuals per year while, in the UK (Fig. 5cfi), HZ incidence is predicted to continuously decrease together with the progress of mass immunization.

Medium-term predictions indicate that an increase of HZ incidence is not a certain fact, but rather seems to depend on the presence or absence of factors which promote a strong boosting intensity (and, as a consequence, a lower HZ susceptibility) and thus may or may not be heavily affected by changes in varicella circulation due to mass immunization. Indeed an increase in HZ incidence occurs in countries where the incidence rate was lower prior to vaccination, possibly due to a higher force of boosting (e.g. Finland), whereas the increase in HZ incidence is minor or absent where the force of boosting was milder (e.g. the UK).

A general remark about the impact of VZV immunization on HZ is that whatever the country specific initial conditions in terms of HZ incidence and VZV force of infection, and despite different assumptions about the degree of success of immunization in the various countries, HZ incidence remain steadily above around 3 per 1000 cases per year and lands on the level of 3 per 1000 after about 60 years of vaccination programs. This surprising convergence result is quantitatively accurate at high immunization levels (as exemplified either by the one dose-100% program, or by the two-doses programs, respectively in the top and bottom rows of Fig. 5). The explanation is that at high levels of immunization the force of boosting, which is the only factor differentiating the HZ epidemiology among the three countries considered, rapidly becomes negligible and the three countries remain exposed only to the equalizing role jointly played by vaccination (generating an identical flow of HZ cases from the vaccine strain the three countries) and by the action of biological HZ parameters, which by hypotheses are identical in the different countries. At lower immunization levels (as exemplified by the middle row in Fig. 5) convergence is only slightly less quantitatively accurate due to the fact that the lower coverage allows some differences in the force of boosting among countries to persist over time.

**Figure 6:**
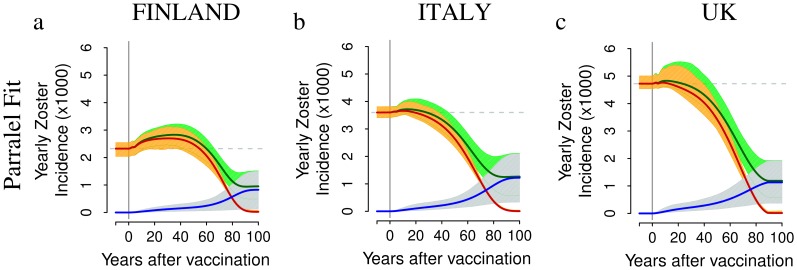
HZ post-vaccination dynamics obtained by adopting the *parallel* fit approach. Top row. Yearly incidence of HZ (average in dark green, 95% CI in light green),of natural HZ – i.e., by HZ cases occurring among unvaccinated individuals that have experienced natural varicella – (average in red,95% CI in orange) and of HZ caused by the vaccine strain (average in blue, 95% CI in light blue) per 1,000 individuals as obtained by adopting the *parallel* fit approach and by simulating the two-dose scenario, which assumes the administration of a first dose to 1 year-old individuals (90% coverage) and a second dose to 5 years-old individuals (80% coverage), in Finland (a) in Italy (b) and in the UK (c). Results are based on 1,000 model realizations.

Our results about the impact of VZV immunization on HZ incidence, e.g. in the UK, are remarkably different from those obtained in previous modeling studies which were dealing with a single country 12,23–28,32. Indeed, by removing the constraint that the duration of CMI and the reactivation rate are the same in all countries (as in our *parallel* fit approach) the HZ incidence is predicted to increase in all countries considered, as illustrated in the two-dose scenario of Fig. 6). When the 70% program is considered instead of the 100% program, the predicted increase in HZ incidence is smaller (e.g. for Finland, see Fig. 5d) due to the smaller impact of vaccination on boosting, but the burden of natural varicella obviously remains larger **(** Fig. 3d).

For at least 50 years after the introduction of the vaccine, the dynamics of HZ incidence are driven by natural HZ, i.e. by HZ cases occurring among unvaccinated individuals that have experienced natural varicella (Fig. 5). This is the consequence of the decline of protective effect of boosting against HZ for unvaccinated individuals as a consequence of the reduction of varicella infected individuals caused by mass VZV immunization. This phenomenon is well illustrated by the ideal scenario of a 100% coverage program, implemented with a *perfect* vaccine with probability of failure equal to zero and conferring permanent immunity against varicella (details in Text S1). For instance, in Finland, HZ incidence is predicted to increase in the short-medium term due to the removal of the protective effect of boosting, and to vanish in the long term due to the gradual removal of all VZV and HZ susceptibles through the perfect protection provided by vaccination. However, this process requires at least the removal, by natural causes, of an entire generation, i.e. more than 70–80 years.

On the other hand, once the population becomes mostly composed of vaccinated individuals and the fraction exposed to natural varicella declines, which occurs after 5–6 decades after the introduction of the immunization program, HZ is strongly reduced by vaccination and attains levels well below those before vaccination, reaching in all countries levels around 2 per 1000 per year. Nonetheless, as already shown in [23], [28], HZ is far from being eliminated, given that the vaccine protects only partially against varicella and HZ. In the long term, HZ incidence is mainly caused by the vaccine strain. For instance, if a coverage of 100% is considered, the latter is expected to slowly increase from the beginning of the immunization program up to around 0.9 per 1000 individuals per year after 100 years of vaccination. The large uncertainty surrounding long term predictions on HZ from the vaccine strain compared to natural HZ is again the consequence of the large uncertainty about vaccine-related parameters.

**Figure 7:**
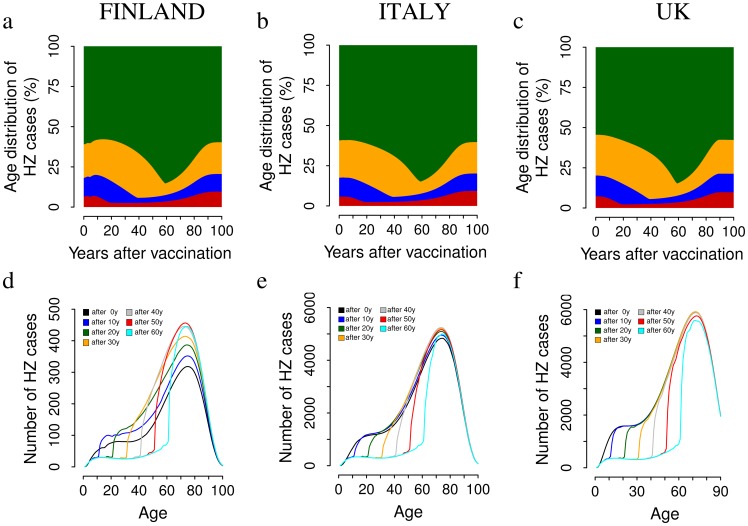
The impact of VZV vaccination on the age distribution of HZ cases. Top row. Average yearly distribution of HZ cases among individuals aged 0–20 (red), 21–40 (blue), 41–60 (orange) and 61+ (green) as predicted by simulating a single vaccine dose administered to 1 year-old infants with 100% coverage in Finland (a) Italy (b) and the UK (c). Results are based on 1,000 model realizations. Bottom row. The absolute HZ incidence by age at various time points following the introduction of the vaccine for Finland (d) Italy (e) and the UK (f).

Predictions of the age distribution of HZ cases for all considered scenarios show that both before and after the introduction of vaccination most of HZ cases occur in the elderly (see Fig. 7 and Text S1). For instance, in Finland, in the absence of immunization, 61% of HZ cases are predicted to occur among 60+ old individuals (59% in Italy and 54% in the UK) while 21% occur among those aged 40–59 (21% in Italy and 25% in the UK). Under the single dose 100% coverage program, the proportion of cases occurring in Finland among individuals older than 60 is predicted to slightly decline initially (first 15 years after the start of immunization) due to the sudden decline in the FOB and consequent increase in zoster across all age groups, and then to sharply increase, up to a maximum of 85%, 55 years after the initiation of the program. Finally, after 100 years of vaccination the age distribution of HZ cases is surprisingly almost *restored* to the pre-vaccination level (see Fig. 7, top row) although the overall number of cases is considerably reduced. This peculiar post-vaccination dynamics is explained in Fig.7 (bottom) which reports the absolute HZ incidence by age at various time points following the introduction of the vaccine. For instance in Finland, in all age groups which are not vaccinated, the absolute incidence of HZ increases due to the sudden decline in the FOB caused by the introduction of the vaccine, which increases the speed at which all initial HZ susceptibles acquire zoster at subsequent times. On the other hand, HZ cases decrease among individuals who have been vaccinated, due to the highly effective role of the vaccine in preventing natural varicella infections (and subsequent HZ development from the wild strain) and dramatically slowing VZV reactivation for vaccinees. The interplay of these two factors yields an increase in the weight of the elderly in the medium term.

Similar results have been obtained for Italy and the UK and for other coverages and schedules considered (see Text S1).

## Discussion

A major factor underlying the stalled introduction of varicella immunization in Europe is the fear, predicted by many modeling efforts [12], [23]–[28], of a HZ boom in the first few decades after the start of vaccination, due to the predicted decline of the protective role of boosting.

We propose an innovative use of the mathematical models applied in the last decade to investigate VZV transmission dynamics and reactivation, with the aim of shedding new light on the controversial effect of varicella immunization on HZ epidemiology from a European, multi-country, perspective. This is motivated by two main departure points. The first one is that, though HZ age-specific incidence curves in different countries have similar shapes, they also show a large differences in scale, which calls for an explanation. For instance, why is HZ incidence about twice as high in the UK compared to Finland at essentially all ages? Recent research has documented that rates of HZ are systematically lower in countries with high VZV transmission [1], [56] (though similar comparisons can be hampered by between-country differences in the surveillance and reporting rates). The second one is that traditional single-country modeling approaches to varicella and HZ, have nicely explained in the past HZ incidence curves in different countries [12], [23]–[28] but when tested in parallel - as done here - they have provided inconsistent estimates for critical HZ parameters, such as CMI duration that, having a biological basis, should not show any significant inter-country variation. To avoid this inconsistency, we have attempted to estimate HZ parameters in a *simultaneous*, multi-country, approach by constraining CMI duration and the VZV reactivation rate to be the same in all countries. This strategy enabled us to identify the main determinant of the large pre-vaccination cross-country variation in HZ scale as being a marked difference in the force of boosting in the various countries. Finally, we have made an effort to appropriately handle the large uncertainty about HZ parameter estimates which derives from the impossibility of observing HZ susceptibility by retaining in our uncertainty analyses all HZ parameter configurations which provided an adequate fit to HZ incidence data.

The model has been used to investigate the impact of different varicella mass immunization programs on HZ epidemiology. These analyses were carried out by taking into account not only the uncertainty surrounding VZV transmission and reactivation, but also the large uncertainty surrounding vaccine-related parameters (i.e. the vaccine take, the duration of vaccine immunity and the currently poor estimates related to HZ development following VZV immunization). The ensuing short and medium term predictions about HZ indicate in a surprisingly robust manner (i.e. characterized by rather narrow prediction bands), that VZV vaccination does not necessarily yield a HZ *boom*. Rather HZ booms are likely to occur where HZ incidence was *low* prior to vaccination because the force of boosting was high (e.g. Finland), whereas no increase in HZ is expected where the force of boosting appears to be low (e.g. the UK). These results are quite different from previous single-country modeling studies unanimously predicting a post varicella vaccination HZ increase [12], [23]–[28].

However, and this is the second central finding of our work, it seems that – in the absence of any specific intervention to prevent HZ [57] - whatever the country specific initial scale of HZ incidence and VZV force of infection, and despite possibly different degrees of success of immunization in the various countries in the short and medium term, HZ incidence can never be lowered below 3 per 1000/year in the first 60 years following immunization. In simple words, this fact, which can only be appreciated in the multi-country approach, means that though after immunization HZ increases in Finland and declines in the UK, the overall HZ incidence predicted in Finland always remains below the one predicted in the UK, and overlaps in the medium-long term. This *convergence* result in HZ patterns suggests the existence of a *natural* post-vaccination equilibrium in HZ incidence which all countries will face in the long term. In some countries, such as Finland, this equilibrium will be approached from a relatively lower pre vaccination incidence (possibly due to a presently higher boosting effect); in some other countries, such as the UK, from a relatively higher prior to vaccination incidence rate (possibly due to milder boosting effect). This convergence finding, which is due to the *equalizing* role of vaccination (and common HZ parameters) seems to be important to consider from an international, e.g. European, policy making viewpoint. Actually the result of convergence in HZ post vaccination dynamics should be proved in a larger number of countries to be considered as a generic phenomenon. However, we feel that the argument that HZ is expected to increase more where HZ is lower and vice-versa which underlies our concept of convergence, is fairly robust. The reason for the robustness of medium-term HZ predictions lies in the fact that essentially all cases of HZ arising in the medium term occur among individuals who acquired varicella before the initiation of the vaccination program and whose numbers are therefore not affected by the uncertainty surrounding vaccine related parameters (which instead cause a large uncertainty about varicella predictions). Finally, in the very long term, the further decline in natural zoster is counter-balanced by vaccine related zoster, and the overall HZ incidence stabilizes at about 1 per 1000 in all countries.

As our predictions show that - both before and after the introduction of vaccination - most of HZ cases occur in individuals older than 60 years, the newly introduced vaccine against HZ has the potential to partly mitigate this *unavoidable* burden of zoster [23]. However, as recently pointed out in [29], [58], the potential coverage of HZ vaccination has to be carefully considered. Indeed a very low uptake (less than 10%), has been reported for the US in the 2008–2009, possibly caused by low perceived risk for HZ [59], [60].

Our results, compared to prior modeling studies, arise due to the multi-country modeling perspective (and indeed do not arise in the traditional single-country perspective). This suggests that multi-country modeling might be an important strategy every time paucity of data yields inconsistent estimates of biological parameters. Specifically, our results are mainly due to the estimated variation in the country-specific force of boosting. Current generation VZV models represent the force of boosting as proportional to the force of varicella infection [12], [23]–[27]. However it is well know that the force of infection is poorly estimated among adults and the elderly, i.e. the groups which are at risk of boosting and HZ. Further research aimed at improving our knowledge of contact patterns and boosting in the elderly would thus be needed.

More in general, mathematical modeling of VZV and HZ is based the Hope-Simpson boosting hypothesis [5].This hypothesis has received some support [11], [12], although in recent times also opposing evidence has been presented [10]. Moreover, surveillance of HZ incidence in sites where mass vaccination is ongoing has shown ambiguous results. Indeed, though there is some favorable evidence of increasing HZ incidence [2], [15], [29], [30], [44], similar increases have been detected also in countries without a relevant history of vaccination [30]. Moreover, sites without HZ increase have also been reported [30]. Our model predictions of the possibility of both increasing and decreasing trends depending on both the scale of HZ incidence prior to immunization and the extent of the force of boosting, may thus explain these apparently ambiguous observations. This suggests potentially useful model-informed strategies for the selection of HZ sentinel sites in post-vaccination regimes aimed at detecting possible symptoms of HZ upsurge. Indeed, our results suggest which are the sites where such increases in HZ incidence are more likely to occur: those where pre-vaccination HZ incidence was low due to a high pre-vaccination force of boosting.

Most of all however, there is a lack of knowledge about CMI duration and boosting. Recent high-level joint immunological-field studies such as [13] represent a first step toward a better understanding of the complex epidemiology of HZ, but more work in this direction is needed. Anyhow we believe that Hope-Simpson’s exogenous boosting hypothesis is for the time being a non-dismissible hypothesis in VZV modeling. Renouncing to this hypothesis would mean to consider the alternative scenario where the appearance of zoster is unrelated to re-exposure to VZV. In this case obviously vaccination wouldn’t increase the risk of progression to HZ, and therefore HZ incidence would decline together with varicella cases in both the medium and long term.

This study aimed at better understanding the impact of VZV mass vaccination on HZ epidemiology. From this perspective, our models may be useful to evaluate further immunization options, such as the use of the HZ vaccine [23], [24]. The predicted impact on varicella is much more uncertain due to the high uncertainty in vaccination parameters. Overall, our results suggest caution when evaluating the introduction of mass vaccination against VZV. First, mass vaccination could lead in the medium-long term to sustained VZV transmission through breakthrough infections, which could contribute to eroding the trust of the public in immunization programs. Second, as average age at infection is predicted to increase significantly, clinical effects of varicella breakthrough on adults and elderly should be carefully studied. Third, our finding of possibly different effects on HZ epidemiology depending on pre-vaccination conditions may have important effects on the overall cost-effectiveness of vaccination and may also question previous findings [24].

### Conclusions

Unlike previous single-country modeling studies [12], [23]–[28], [32], the proposed multi-country perspective shows, under different vaccination scenarios, that an increase in HZ incidence is not a certain fact, but rather seems to depend on the presence or absence of factors promoting a strong boosting intensity and that may, or may not, be heavily affected by changes in varicella circulation due to the introduction of mass immunization programs.

These findings might provide an explanation for the ambiguous empirical evidences about the increases of HZ in those sites where mass varicella vaccination is ongoing [30]. In particular, they suggest which are the sites where such increases in HZ incidence are more likely to occur: those where pre-vaccination HZ incidence was low due to a high pre-vaccination force of boosting. Therefore, these findings supply potentially useful strategies for the selection of HZ sentinel sites aimed at detecting possible symptoms of HZ upsurge as a consequence of mass immunization.

Moreover, though following VZV immunization HZ is predicted to increase in Finland and to decline in the UK, the overall HZ incidence in Finland always remains below the corresponding UK figure, and overlaps to it in the medium-long term. This finding suggests an *equalizing* role of vaccination which is important to consider from an international, e.g. European, policy making viewpoint.

## Supporting Information

Text S1 **Details on model formulation and parameterization, and additional results.** In this appendix a detailed description of the mathematical model considered in this manuscript is included. Model parameterization, and additional results are discussed.
(PDF)
