# Supplementary material for: Perspectives on the Impact of Varicella Immunization on Herpes Zoster. A Model-Based Evaluation from Three European Countries
Source: PLoS One. 2013 Apr 17;8(4):e60732. doi: 10.1371/journal.pone.0060732 (PMC3629254; doi:10.1371/journal.pone.0060732)
Supplement: Text S1 — Details on model formulation and parameterization, and additional results. In this appendix a detailed description of the mathematical model considered in this manuscript is included. Model parameterization, and additional results are discussed. (PDF) [file pone.0060732.s001.pdf]

Perspectives on the impact of varicella immunization on herpes zoster. A model-based evaluation from three European countries

Piero Poletti[1,2], Alessia Melegaro[2], Marco Ajelli[1], Emanuele del Fava[3], Giorgio Guzzetta[1], Luca Faustini[4], Giampaolo Scalia Tomba[5], Pierluigi Lopalco[6], Caterina Rizzo[7], Stefano Merler[1] & Piero Manfredi[8]

[1] Center for Information Technology, Bruno Kessler Foundation, Trento, Italy,
[2] DONDENA Centre for Research on Social Dynamics, Bocconi University, Milan, Italy,
[3] Center for Statistics, University of Hasselt, Diepenbeek, Belgium,
[4] ISTAT Regional Office for Tuscany, Firenze, Italy,
[5] Department of Mathematics, University of Rome-Tor Vergata, Italy,
[6] European Centre for Disease Prevention and Control, Stockholm, Sweden,
[7] National Center for Epidemiology Surveillance and Health Promotion, Istituto Superiore di Sanitá, Rome, Italy,
[8] Department of Statistics and Mathematics Applied to Economics, University of Pisa, Italy,

Text S1

Contents

Contents 2

1 The model 2
1.1 VZV transmission model . . . 2
1.2 Vaccination . . . 3
1.3 Age transitions . . . 3
1.4 Vaccine administration . . . 4

2 Model parametrization 5
2.1 Estimation of varicella transmission rates and basic reproduction numbers . . . 5
2.2 Simultaneous estimation of HZ parameters . . . 6
2.3 Robustness of the simultaneous approach . . . 7

3 Additional results **7**
3.1 The estimates of HZ parameters 7
3.2 The impact of mass VZV immunization 7

1 The model

1.1 VZV transmission model

The model considers a population subdivided in 100 yearly age groups closed to migrations and in a stationary situation i.e., the total numbers and the age distribution of the individuals in the population do not change over time. Each year a constant number B of newborn individuals (equally distributed during the year and appearing at a daily rate B/365) enter the population and B individuals leave the population due to mortality following age-specific mortality rates.

Individuals born from mothers who are immune to, or infected from VZV, inherit VZV maternal antibodies (M), while births from susceptible and exposed individuals are assumed to be susceptible (VS). In fact, there might be a non negligible fraction of newborn from susceptible mothers, especially in post vaccination epochs when the average age of infection is expected to increase. This issue has been addressed by computing the fraction of individuals who are both susceptible or exposed and eligible for having a baby on the basis of available age-specific fertility and mortality rates and sex proportions in the population, as reported by the Eurostat database [**?**].

Maternal antibodies are assumed to decay at a constant rate μ with an average duration of protection of $1/\mu$; VZV susceptible individuals (VS) are exposed to an age-specific force of infection (FOI) $\lambda(a)$. In absence of vaccination, the FOI is defined as follows:

$$\lambda(a) \quad = \quad \sum_{j=0}^{100} q \; C(a,j) \left(\frac{VI(j)}{N(j)} + i_{HZ} \frac{(ZI(j)}{N(j)} \right) \tag{1}$$

where q is an age-independent transmission coefficient; $C(a,j)$ is the age-specific contact matrix whose entries describe the average numbers of different persons in age group j encountered by an individual belonging to age group a per unit of time; $VI(j)$ and $ZI(j)$ are the number of varicella and HZ infected individuals of age j respectively, i_{HZ} is the relative VZV infectiousness associated to HZ compared to varicella cases with $0 < i_{HZ} < 1$, and $N(j)$ is the number of individuals of age j. Exposed individuals (VE) become infective after an average latency period of $1/\omega$; infective individuals (VI) recover after an average infective period of $1/\gamma$, developing lifelong immunity to varicella and a temporary protection against HZ (VR). Cell mediated immunity (CMI), gained during the primary varicella infection and protecting against HZ, exponentially decays at a constant rate δ, so that after an average period of $1/\delta$, varicella recovered individuals (VR) become HZ susceptible (ZS). HZ susceptible have two possibilities:

- they can boost their CMI by re-exposure to infectious cases reacquiring protection against HZ (VR) at a "force of boosting" (FOB) $z\lambda$, which is assumed to be proportional to the FOI, through an age independent parameter $z < 1$.
- they can progress to HZ disease (ZI), at an age-specific VZV reactivation rate defined as $\rho(a) = \psi e^{-\phi a} + \pi a^{\eta}$ (with $\psi, \phi, \pi, \eta > 0$) [**?**].

HZ infected individuals (ZI) contribute to the varicella FOI λ for an average infective period of $1/\alpha$ on average; afterwards, they become permanently immune to HZ disease.

1.2 Vaccination

A proportion P of vaccinated individuals suffer an initial vaccine failure and remains susceptible, while the complementary fraction of vaccinated individuals $1-P$ has a fixed "take" probability T to acquire temporary protection against VZV infection and a lifelong partial protection against HZ. Vaccine protected individuals lose immunity against varicella at a waning rate W. Vaccinated individuals who suffer a secondary failure (a fraction $(1-T)(1-P)$) and vaccine protected individuals whose immunity against varicella has waned are exposed to the natural FOI, but are assumed to acquire a milder infection commonly referred as "breakthrough varicella" which will be less infectious compared to natural ones. Vaccinees can develop HZ after breakthrough varicella or directly from vaccine strain. The mechanism of varicella and HZ development among vaccinees is the same as the one described for individuals who experience natural varicella infection. However the reactivation rate at which vaccinated HZ susceptibles develop HZ is lower ($\chi\rho(a)$ instead of $\rho(a)$ where $\chi < 1$).

The post-vaccination FOI becomes:

$$\lambda(a) \;=\; \sum_{j=0}^{100} q\, C(a,j) \left(\frac{VI(j)}{N(j)} + i_{BV}\frac{VBI(j)}{N(j)} + i_{HZ}\frac{(ZI(j)+ZBI(j)+ZVI(j))}{N(j)} \right) \tag{2}$$

where $VBI(j)$ is the number breakthrough varicella infected individuals of age j; $ZBI(j), ZVI(j)$ are the number of HZ infected individuals of age j occurring among vaccinated individuals after breakthrough infection and after vaccination only respectively; i_{BV} is the relative VZV infectiousness associated to breakthrough varicella infections compared to natural varicella infections with $0 < i_{BV} < 1$.

1.3 Age transitions

The proposed model is deterministic and age-structured according to the "Realistic Age Structure" (RAS) approach [?]. Transitions between age groups are assumed to occur simultaneously for all individuals in a specific day of the year, mimicking the natural discrete transition of school groups due to the progression of school careers. Specifically, individuals belong to an age group for an entire (school) year and, in a specific day of the year (when school grades begin), all individuals having age a, are assumed to enter age group $a+1$. This assumption allows the model to account for sudden changes in contact patterns by age caused by school recruitment as those experienced by individuals when they start to attend primary school. The RAS discretization of age transitions is applied to all age classes, thereby introducing an approximation for those individuals that do not attend schools. However, contacts among age classes not related to school attendance are usually driven by interactions, as those occurring in workplaces and households, that are less dependent on the way how age transitions occur.

1.4 Vaccine administration

As for age transitions, vaccine administration is incorporated in the model as discrete events at the beginning/end of each year, when individuals age. Routine immunizations are assumed to be administered in correspondence of birthdays only. In all scenarios considered, the first vaccine dose is administered in correspondence of the first birthday, and the second dose at the fifth birthday. We assume that natural immunity prevails on vaccine immunity and that individuals can experience only one episode of varicella and one episode HZ throughout their life. Thus, the first dose of varicella vaccine is assumed to be administered to non vaccinated varicella susceptible individuals, while the second dose is assumed to be administered to individuals who already received a first dose but are still susceptible to varicella. If individuals receive a second dose of varicella vaccine, the responders (a fraction $T(1-P)$ of vaccinated individuals) become vaccine protected. Those who do not respond to the second dose remain in whatever compartment they were already in.

Besides age transitions and vaccine administrations, all remaining processes occur continuously throughout the school year, and they are therefore represented through ordinary differential equations.

The mathematical representation of the model is therefore given by a sequence of 1-year cycles, represented through a set of ODEs describing the dynamics of infection and mortality within the year, and by a discrete aging/vaccination map, which reallocates individuals at the end of each year to their new age groups and, if they have been vaccinated, to the vaccinated classes. A fully mathematically correct representation of the RAS model, though in a simplistic case, can be found in [**?**]. Within the year the VZV epidemiological dynamics for individuals of age a is defined by the following ODEs system where the dot denotes the time derivative $\frac{d}{dt}$:

$$
\begin{cases}
\dot{M}(a) &= -\mu\ M(a) - \nu(a)\ M(a) \\
\dot{VS}(a) &= \mu\ M(a) - \lambda(a)\ VS(a) - \nu(a)\ VS(a) \\
\dot{VE}(a) &= \lambda(a)\ VS(a) - \omega\ VE(a) - \nu(a)\ VE(a) \\
\dot{VI}(a) &= \omega\ VE(a) - \gamma\ VI(a) - \nu(a)\ VI(a) \\
\dot{VR}(a) &= \gamma\ VI(a) - \delta\ VR(a) + z\lambda(a)\ ZS(a) - \nu(a)\ VR(a) \\
\dot{ZS}(a) &= \delta\ VR(a) - z\lambda\ ZS(a) - \rho(a)\ ZS(a) - \nu(a)\ ZS(a) \\
\dot{ZI}(a) &= \rho(a)\ ZS(a) - \alpha\ ZI(a) - \nu(a)\ ZI(a) \\
\dot{ZR}(a) &= \alpha\ ZI(a) - \nu(a)\ ZR(a) \\
\dot{VP}(a) &= -W\ VP(a) - \delta\ VP(a) - \nu(a)\ VP(a) \\
\dot{VBS}(a) &= W\ VP(a) - \lambda(a)\ VBS(a) - \nu(a)\ VBS(a) \\
\dot{VBE}(a) &= \lambda(a)\ VBS(a) - \omega\ VBE(a) - \nu(a)\ VBE(a) \\
\dot{VBI}(a) &= \omega\ VBE(a) - \gamma\ VBI(a) - \nu(a)\ VBI(a) \\
\dot{VBR}(a) &= \gamma\ VBI(a) - \delta\ VBR(a) + z\lambda(a)\ ZBS(a) - \nu(a)\ VBR(a) \\
\dot{ZBS}(a) &= \delta\ VBR(a) - z\lambda(a)\ ZBS(a) - \chi\rho(a)\ ZBS(a) - \nu(a)\ ZBS(a) \\
\dot{ZBI}(a) &= \chi\rho(a)\ ZBS(a) - \alpha\ ZBI(a) - \nu(a)\ ZBI(a) \\
\dot{ZBR}(a) &= \alpha\ ZBI(a) - \nu(a)\ ZBR(a) \\
\dot{ZVS}(a) &= \delta\ VP(a) - \chi\rho(a)\ ZVS(a) - \nu(a)\ ZVS(a) \\
\dot{ZVI}(a) &= \chi\rho(a)\ ZVS(a) - \alpha\ ZVI(a) - \nu(a)\ ZVI(a) \\
\dot{ZVR}(a) &= \alpha\ ZVI(a) - \nu(a)\ ZVR(a)
\end{cases} \tag{3}
$$

where $\nu(a)$ is the age-specific mortality rate. VBS, VBE, VBI, VBR denote vaccinated individuals that are breakthrough susceptible, exposed, infective and recovered respectively. Classes associated to the progress in HZ disease after breakthrough are ZBS, ZBE, ZBI denoting HZ susceptible, infective and recovered individuals respectively. Similarly classes associated to the progress in HZ disease from vaccine strain are ZVS, ZVI, ZVR. The full list of model parameters and their literature sources are given in Table S1.

2 Model parametrization

2.1 Estimation of varicella transmission rates and basic reproduction numbers

Age-specific contact matrices for each country considered were taken from Fumanelli et al. [?]. These contact matrices were computed by an approach similar to the one introduced in [?] but further developed to derive social contact structures for several European countries. Briefly, a synthetic population of agents, each one corresponding to an individual in the real population, is generated by using highly detailed routine socio-demographic data [?], such as

household distributions by type, size and age of members, school sizes for the various school levels, workplace sizes and employment rates by age, and the age distribution of the general population. From this synthetic population, age-specific contact matrices $C(i, j)$ were computed for each country considered.

The varicella transmission rate per social contact q (see Eq. 1) is estimated for each country conditionally on the contact matrix $C(i, j)$, by maximizing the likelihood of VZV seroprevalence data through a simple age-structured SIR model at equilibrium where the contribution of HZ to the force of infection is assumed negligible. The latter assumption is based on the observation that HZ is rarer compared to varicella, less infectious, and it occurs preferentially in older ages, when social contacts with susceptibles individuals are much less frequent compared to younger ages. This assumption allows to estimate VZV transmission regardless the mechanisms governing HZ development.

Our analysis gives q estimates of 0.142 (95% CI 0.137,0.148) in Italy, 0.283 (95% CI 0.264,0.304) in Finland and 0.185 (95% CI 0.176,0.194) in the UK. From the estimates of q the varicella basic reproduction number R_0, which is defined as the number of secondary infections caused by a single infective individual in a fully susceptible population during his/her whole infective period [**?**], is computed as the dominant eigenvalue of the country-specific generation matrices $qC(i, j)/\gamma$ [**?**]. Country-specific distributions of bootstrap estimates of R_0 are displayed in Fig. S1.

2.2 Simultaneous estimation of HZ parameters

HZ parameters have been estimated simultaneously by pooling data from the three countries considered and by constraining intrinsically biological quantities to be the same in all countries. The choice of the countries included in this analysis is based on the availability of varicella age specific seroprevalence profile, HZ age specific incidence and contact synthetic matrices.

Specifically, we assume that the average duration of CMI after primary VZV infection (i.e., $1/\delta$) and the age-dependent VZV reactivation rate (i.e., parameters ψ, ϕ, π, η driving $\rho(a)$) are the same in all countries. On the other hand we let the scaling factor z of CMI boosting to be country-specific, in order to reflect for the larger uncertainty in the FOI, and therefore in the FOB, at higher ages. Indeed, as it is well known, the force of infection is poorly estimated in the region where the prevalence curve flattens and, more generally, data related to varicella primary infection do not supply sufficient information for reliably estimating the re-exposure to VZV for adults and the elderly. Moreover, this parameter is not a purely biological parameter but may depend also on social factors and thus are expected to be different between countries.

Parameters configurations driving HZ development have been obtained through the following bootstrap procedure. An initial parameters configuration is chosen by minimizing simultaneously the mean square error between the observed and the predicted HZ incidence for the three different countries. For each country, residuals between the fitted and the observed HZ incidence were computed and 1,000 new data-sets were simulated by adding to each point of the best fit an error uniformly sampled from the array of residuals previously computed. Therefore 1,000 parameters configurations have been obtained by fitting the model against each of the 1,000 simulated data-set.

Obtained estimates are shown in Table S2.

2.3 Robustness of the simultaneous approach

The robustness of predictions based on the approach adopted has been assessed by comparing our results with those obtained through alternative approaches. If the case of perfect boosting is considered ($z = 1$ for all countries i.e., FOB equal to FOI), the model fails to reproduce the country specific HZ incidences (Fig. S2), unless we relax our principal hypothesis that the duration of CMI and the reactivation rate are the same in all countries. In the latter case, an advantage of the multi-country approach is that we could show that separate fitting for each country (what we call "parallel"), though is able to well reproduce the HZ incidences in each country, yields to strongly inconsistent inter-country estimates of parameters having a biological basis. For instance, this is the case of the average CMI duration (see Fig. S3).

3 Additional results

3.1 The estimates of HZ parameters

Here we report further results on HZ parameters estimates. The performed investigation reveals that, on average, the reactivation rate (Fig. S4) is predicted to decline from birth to about the age 30 years and starting increasing monotonically thereafter. This is consistent with the idea that immune competence may be not completely developed in young children and that it may decrease with age. The average duration of CMI is estimated to be in the range of 60-100 years and HZ susceptibility is characterized by a peak around the age of 60 years (see Fig. 2 in the main text). The predicted age distribution of boosting episodes (Fig. S5) is similar among countries and it is characterized by two peaks, the first one at ages 5-10 years and the second one at ages 20-50 - this shape is determined by contacts of infected children with siblings and/or schoolmates in the first case and with parents in the second. The age distribution of boosting episodes essentially determine the profile of HZ susceptibility by age: this is clearly visible in Finland (Fig. 1 in the main text), where a strong intensity of boosting is predicted.

3.2 The impact of mass VZV immunization

The impact of VZV mass immunization on varicella and HZ over time has been investigated for Finland, Italy and the UK for different programs, in terms of coverage and schedules. Main scenarios are displayed and discussed in the main text, while some scenarios based on alternative uptakes and schedules are displayed in Fig. S6, Fig. S7, Fig. S8, Fig. S9. Specifically, the predicted yearly varicella and HZ incidences by assuming a single dose administered to 1 year old infants with 90% and 80% vaccine uptake are displayed in Fig. S6 and Fig. S7. Predictions on the age distribution of varicella and HZ cases for the single dose program with coverages 70%, 80%, 90% are displayed in Fig. S8 and Fig. S9.

Fig. S10 shows the impact of mass immunization on the force of boosting when a single dose administered to 1 year old infants with 100% coverage is assumed. As discussed in the main text, a dramatic decline of yearly boosting incidence is expected under VZV vaccination. For example, a decline of 78% in Finland, 97% in Italy and 94% in UK is expected when 100% coverage is considered.

The ideal scenario of 100% coverage program with a perfect vaccine having failure probability equal to zero and conferring permanent immunity against varicella is displayed in Fig. S11. In this illustrative scenario neither vaccine waining immunity nor vaccine failure are considered (i.e., $W = 0, T = 1$). In Finland, HZ incidence is predicted to increase in the short-medium term, due to the removal of the protective effect of boosting, and to vanish in the long term, due to the gradual removal of all VZV and HZ susceptibles through the perfect protection provided by vaccination. On the other hand, in the UK, where the predicted role of boosting in absence of vaccination is lower, HZ incidence is predicted to continuously decrease together with the progress of the mass immunization program.

In the proposed model individuals who did not suffer of primary vaccine failure can develop HZ, though at a lower rate. Our simulations consider a probability of secondary vaccine failure T in the range of $(0.8, 1)$ and a reduction factor χ of the reactivation rate for vaccinated individuals in the range of $(1/12, 1/4)$ (see Table S1). Given the relevant role played by HZ cases occurring among vaccinees in the long term, the HZ incidence has been investigated by assuming a larger uncertainty on these vaccine parameters values. Specifically, the illustrative scenario of a single dose administered to 1 year old infants with 100% of coverage is investigated by considering T in the range of $(0.7, 1)$ and χ in the range of $(0.1, 0.8)$ (see Fig. S12). The large uncertainty embedded on vaccine parameters results in a large uncertainty in the predictions on HZ from the vaccine strain compared to natural HZ.

Table S1: **Parameters based on the literature**

Parameter	Interpretation	Source	Value
$1/\mu$	Average duration of the maternal antibodies protection	[?]	6 months ‡
$1/\omega$	Average duration of the latency period	[?]	14 days ‡
$1/\gamma$	Average duration of the varicella infectivity period	[?]	7 days ‡
$1/\alpha$	Average duration of the HZ infectivity period	[?]	7 days ‡
C_{ij}	Age specific contact matrices (different for each countries)	[?]	based on census data
i_{BV}	Relative VZV infectiousness of breakthrough varicella cases	[?]	0.5 ‡
i_{HZ}	Relative VZV infectiousness of HZ cases	[?]	0.05 ‡
T	Vaccine take (probability)	assumed	$U(0.8, 1)$†
$1/W$	Average duration of vaccine waining immunity	assumed	$U(0, 200)$ years †
χ	Reduction factor of the reactivation rate for vaccinees	assumed	$U(1/12, 1/4)$ †

‡ Parameters' values taken as constant based on reliable literature estimates.
† Parameters' values are sampled from uniform distributions $U(a, b)$ to account for the large uncertainty of the estimates available in the literature [**?** **?** **?** **?** **?** **?**]

Table S2: **Parameters estimated by direct fitting of the model**

Parameter	Interpretation	Source	Value
q_{FI}	Adjusting factor for contacts relevant for the VZV transmission	fitted against VZV seroprevalence	0.283 (95% CI 0.264,0.304)
q_{IT}	Adjusting factor for contacts relevant the VZV transmission	fitted against VZV seroprevalence	0.142 (95% CI 0.137,0.148)
q_{UK}	Adjusting factor for contacts relevant for the VZV transmission	fitted against VZV seroprevalence	0.185 (95% CI 0.176,0.194)
z_{FI}	Adjusting factor for contacts relevant for the boosting of CMI	fitted against HZ incidence	0.92 (95% 0.80,0.99)
z_{IT}	Adjusting factor for contacts relevant for the boosting of CMI	fitted against HZ incidence	0.41 (95% 0.23,0.74)
z_{UK}	Adjusting factor for contacts relevant for the boosting of CMI	fitted against HZ incidence	0.032 (95% 0.0037,0.0837)
$1/\delta$	Average duration of the CMI (years)	fitted against HZ incidence	79.74 (95% 64.87,106.98)
ψ	parameter defining the reactivation rate $\rho(a) = \psi e^{-\phi a} + \pi a^{\eta}$	fitted against HZ incidence	0.066 (95% 0.015,0.129)
π	parameter defining the reactivation rate $\rho(a) = \psi e^{-\phi a} + \pi a^{\eta}$	fitted against HZ incidence	0.071 (95% 0.020072,0.12006)
ϕ	parameter defining the reactivation rate $\rho(a) = \psi e^{-\phi a} + \pi a^{\eta}$	fitted against HZ incidence	5.40e-07 (95% 2.79e-08,7.28e-07)
η	parameter defining the reactivation rate $\rho(a) = \psi e^{-\phi a} + \pi a^{\eta}$	fitted against HZ incidence	2.71 (95% 2.53,3.41)

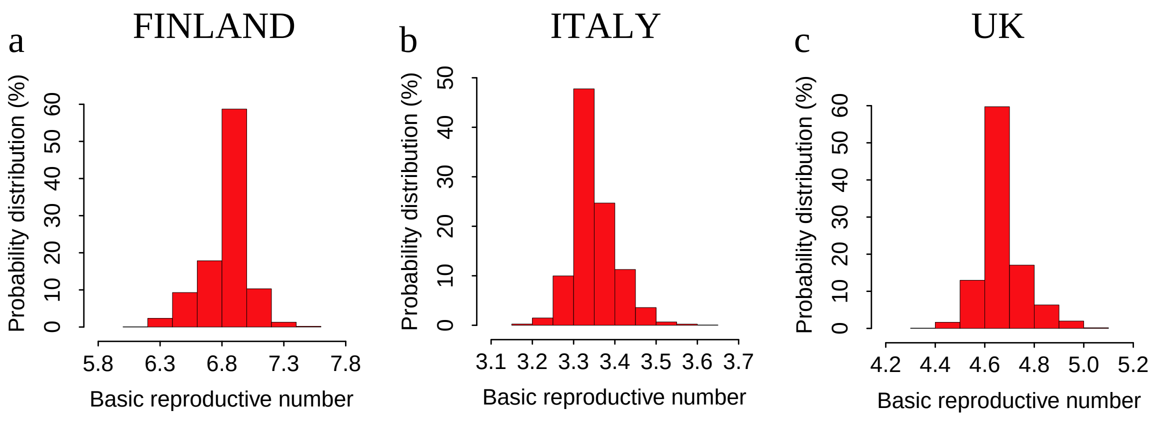

Figure S1: **Estimated basic reproductive number (R_0) in the different countries.** Probability distribution of R_0 as obtained by fitting age-specific serological data for Finland (a) Italy (b) and the UK (c). Results are based on 1,000 model realizations.

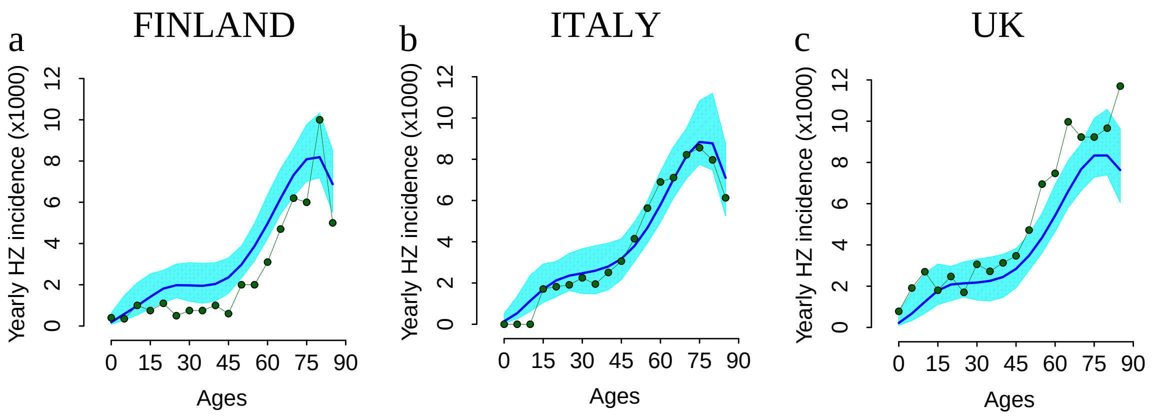

Figure S2: **Alternative assumption on HZ development: the boosting of CMI is assumed to be the same in all countries and the force of boosting is assumed equal to the force of infection (i.e., $z = 1$).** The model fails to reproduce country specific HZ incidence data contemporaneously in all countries. Comparison between the observed HZ incidence among different age classes (green) and the average HZ incidence as predicted by the model (average in blue, 95% CI in cyan) for Finland (a), Italy (b) and the UK (c). Results are based on 1,000 model realizations.

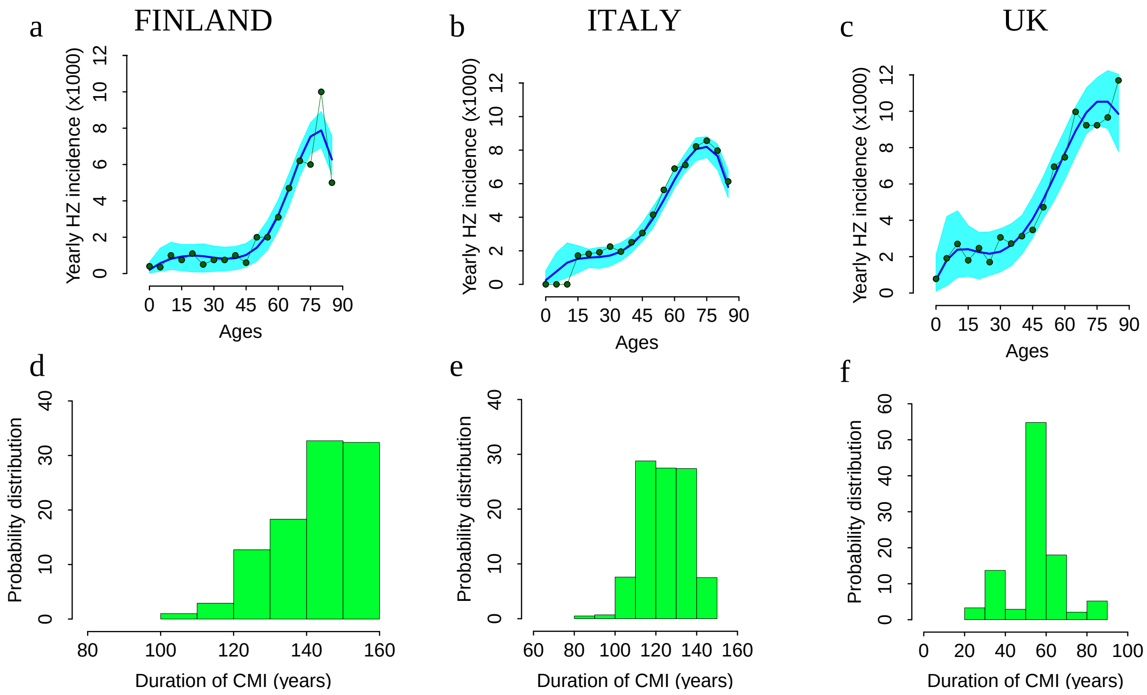

Figure S3: **Alternative assumption on HZ development: HZ incidence data are fitted to the model separately for each country. Top row:** Comparison between the observed HZ incidence among different age classes (green) and the average HZ incidence as predicted by the model (average in blue, 95% CI in cyan) for Finland (a), Italy (b) and the UK (c). **Bottom row:** Probability distribution of the predicted average duration of CMI in Italy (a), Finland (b) and the UK (c). Estimates of parameters regulating biological process like the rate of loss of CMI result remarkably different between countries: this is not biologically sound. Results are based on 1,000 model realizations.

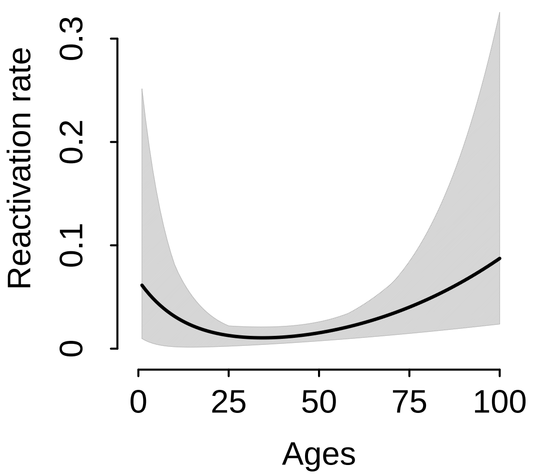

Figure S4: **VZV reactivation rate.** Age specific VZV reactivation rate obtained by simultaneously fitting observed zoster incidence in the three different countries (average in black, 95% CI in gray). Results are based on 1,000 model realizations.

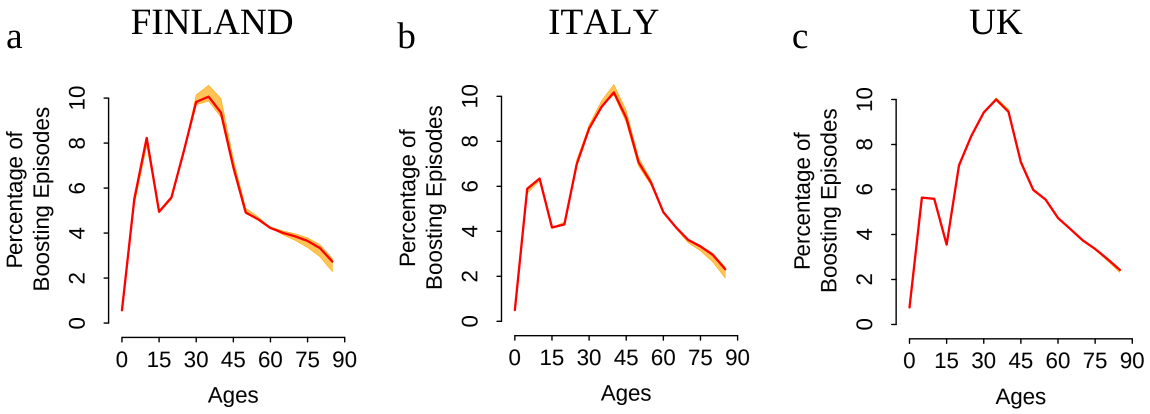

Figure S5: **Pre-vaccination boosting.** Distribution of boosting episodes by age as predicted by the model (average in red, 95% CI in orange) for Finland (a), Italy (b) and the UK (c). Results are based on 1,000 model realizations.

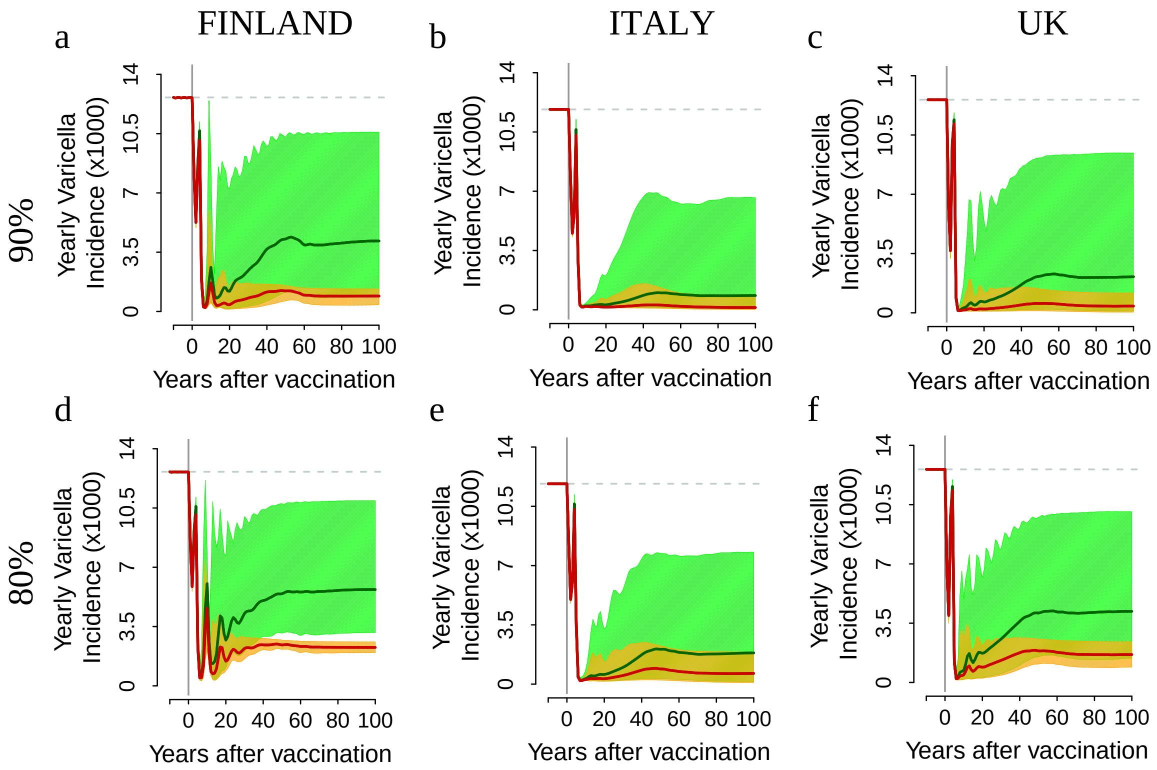

Figure S6: **The impact of VZV vaccination on varicella for other coverages. Top row:** Yearly incidence of varicella (average in dark green, 95% CI in light green) and of natural varicella (average in red, 95% CI in orange) per 1,000 individuals as predicted by simulating a single vaccine dose administered to 1 year-old infants with 90% coverage in Finland (a) in Italy (b) and in the UK (c). **Bottom row:** As the top row but obtained by considering 80% coverage in Finland (d) in Italy (e) and in the UK (f).

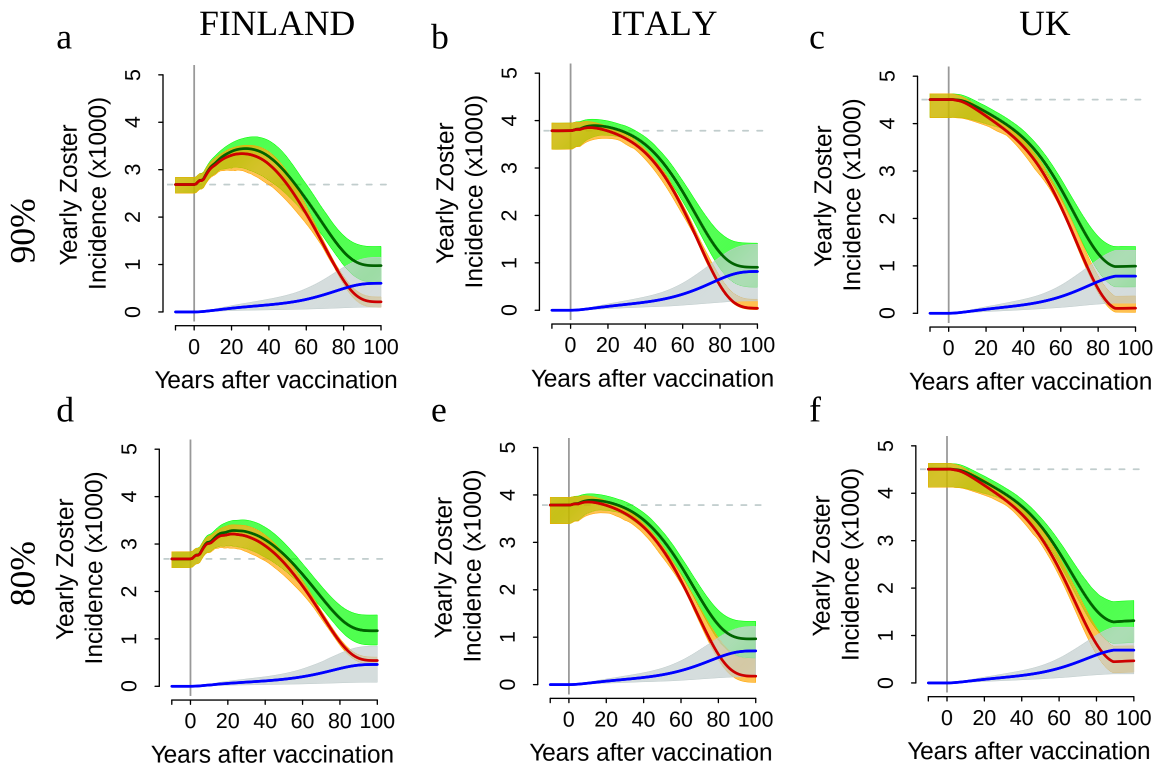

Figure S7: **The impact of VZV vaccination on zoster for other coverages. Top row:** Yearly incidence of HZ (average in dark green, 95% CI in light green), of natural HZ i.e., by HZ cases occurring among non-vaccinated individuals that have experienced natural varicella (average in red, 95% CI in orange) and of HZ caused by the vaccine strain (average in blue, 95% CI in light blue) per 1,000 individuals as predicted by simulating a single vaccine dose administered to 1 year-old infants with 90% of coverage in Finland (a) in Italy (b) and in the UK (c). **Bottom row:** As the top row but obtained by considering 80% coverage in Finland (d) in Italy (e) and in the UK (f).

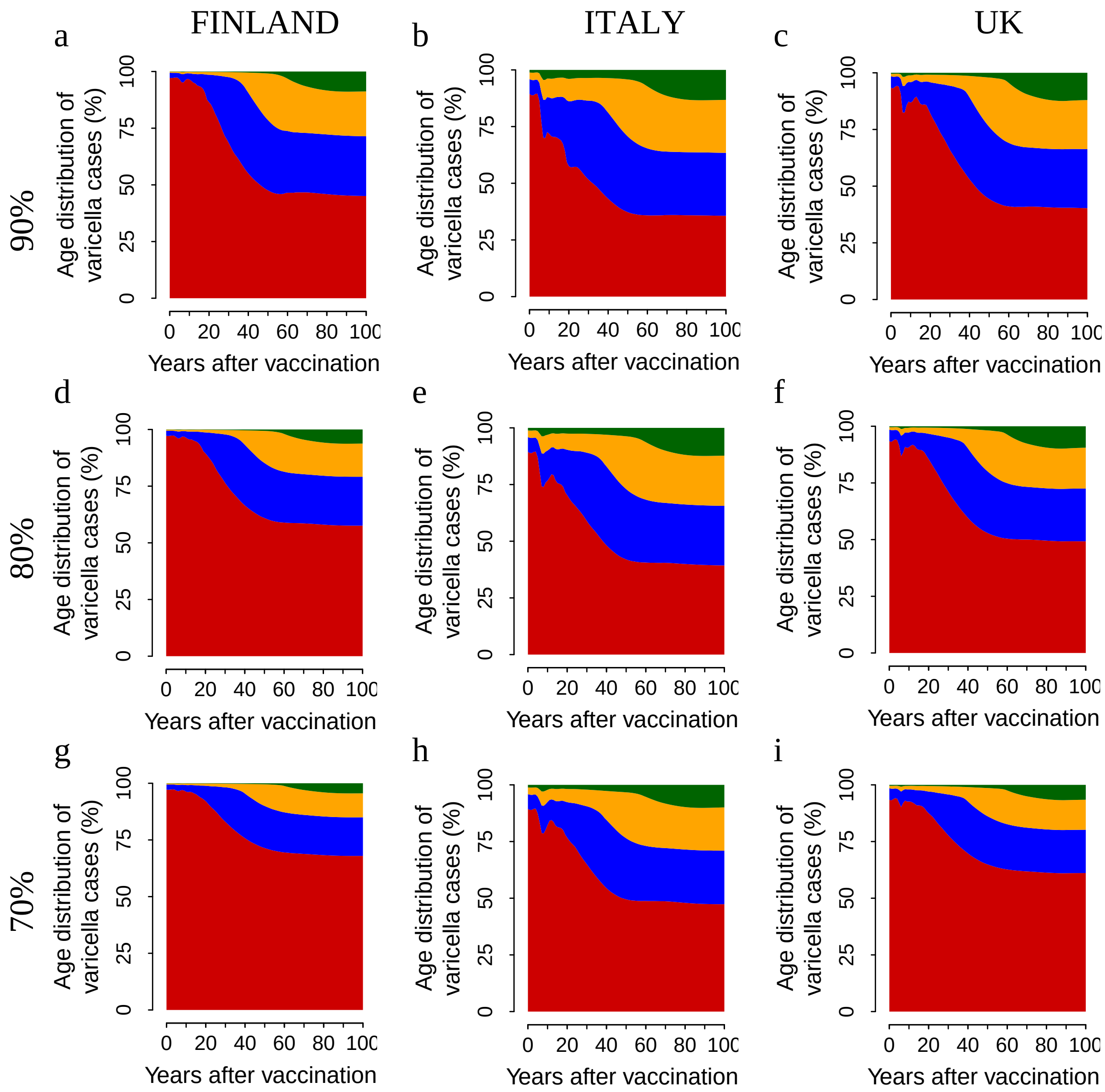

Figure S8: **The impact of VZV vaccination on the age distribution of varicella cases. Top row.** Yearly distribution of varicella cases in individuals aged 0-20 (red), 21-40 (blue), 41-60 (orange) and 61+ (green) as predicted by simulating a single vaccine dose administered to 1 year-old infants with 90% of coverage in Finland (a) Italy (b) and the UK (c). Results are based on 1,000 model realizations. **Mid row:** As the top row but obtained by considering 80% coverage in Finland (d) in Italy (e) and in the UK (f). **Bottom row:** As the top row but obtained by considering 70% coverage in Finland (g) in Italy (h) and in the UK (i).

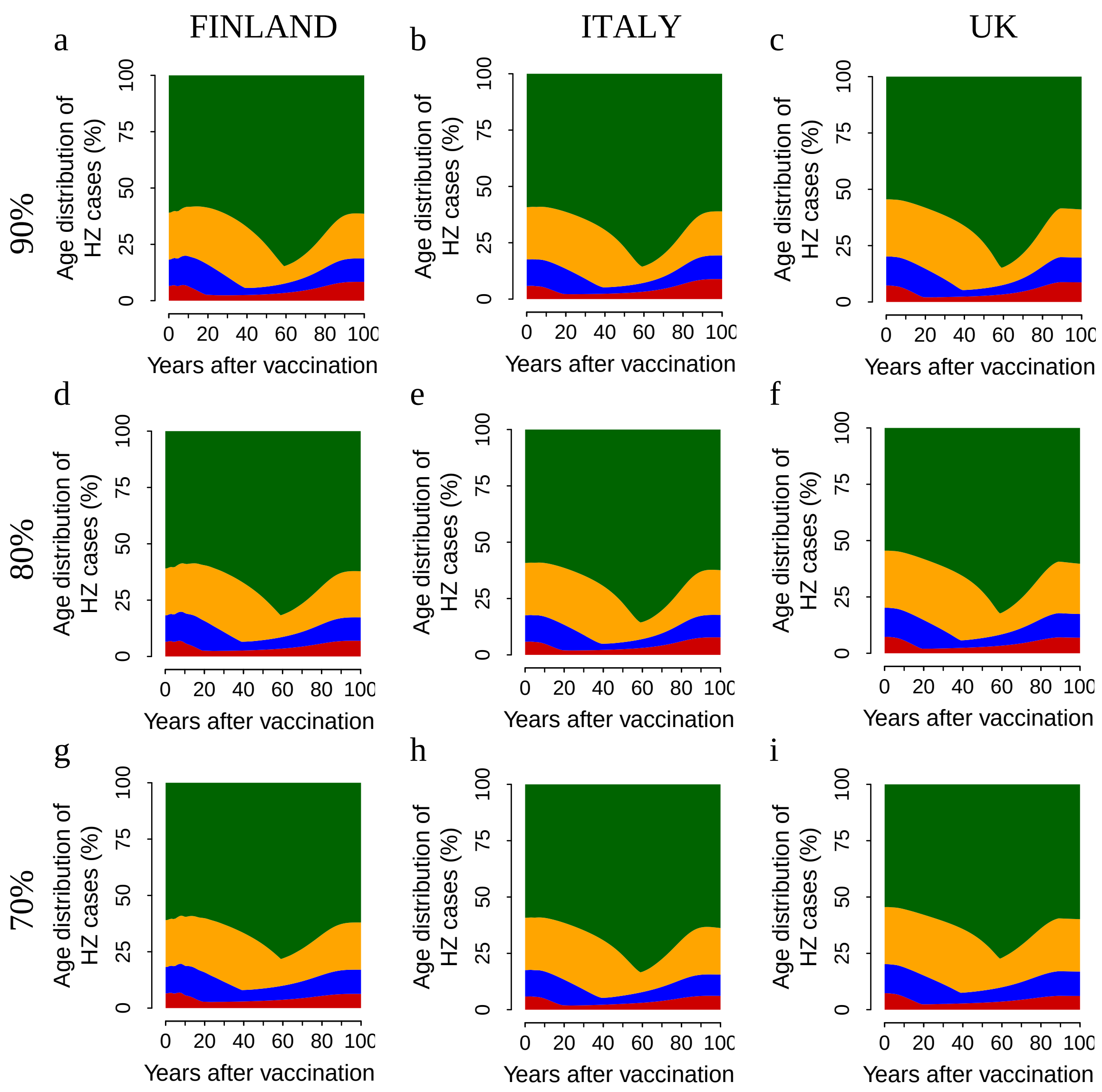

Figure S9: **The impact of VZV vaccination on the age distribution of HZ cases. Top row.** Yearly distribution of HZ cases in individuals aged 0-20 (red), 21-40 (blue), 41-60 (orange) and 61+ (green) as predicted by simulating a single vaccine dose administered to 1 year-old infants with 90% of coverage in Finland (a) Italy (b) and the UK (c). Results are based on 1,000 model realizations. **Mid row:** As the top row but obtained by considering 80% coverage in Finland (d) in Italy (e) and in the UK (f). **Bottom row:** As the top row but obtained by considering 70% coverage in Finland (g) in Italy (h) and in the UK (i).

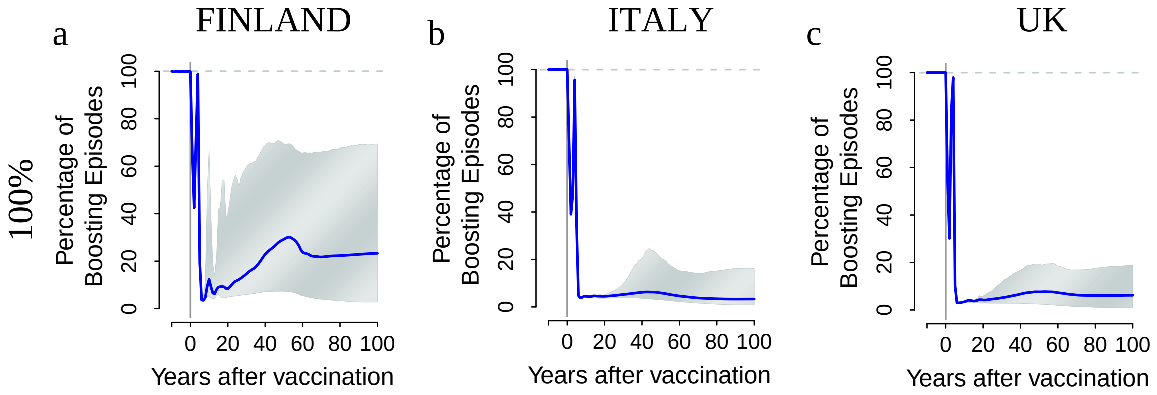

Figure S10: **The impact of vaccination on boosting.** Fraction of yearly incidence of boosting episodes on the yearly incidence of boosting episodes predicted at the pre-vaccination equilibrium (average in blue, 95% CI in light blue) per 1,000 individuals as predicted by simulating a single vaccine dose administered to 1 year-old infants with 100% coverage in Finland (a), in Italy (b) the UK (c).

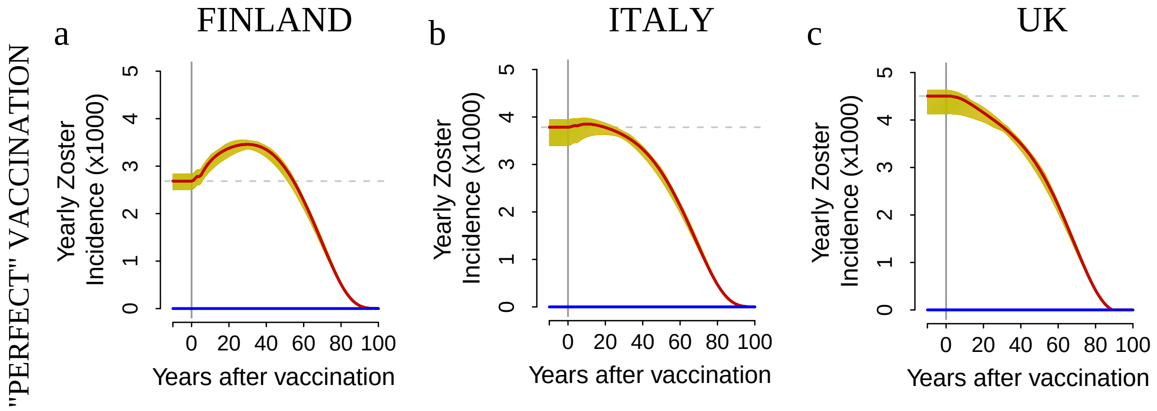

Figure S11: **The ideal scenario of perfect vaccine.** Yearly incidence of HZ (average in dark green, 95% CI in light green), of natural HZ i.e., by HZ cases occurring among non-vaccinated individuals that have experienced natural varicella (average in red, 95% CI in orange) and of HZ caused by the vaccine strain (average in blue, 95% CI in light blue) per 1,000 individuals as predicted for Finland (a), Italy (b) and the UK (c) by simulating a a single dose administered to 1 year old infants with 100% of coverage and by assuming $W = 0$ i.e., no vaccine waining immunity, and the take probability equal to 1 ($T = 1$ i.e., probability of secondary failure is equal to zero).

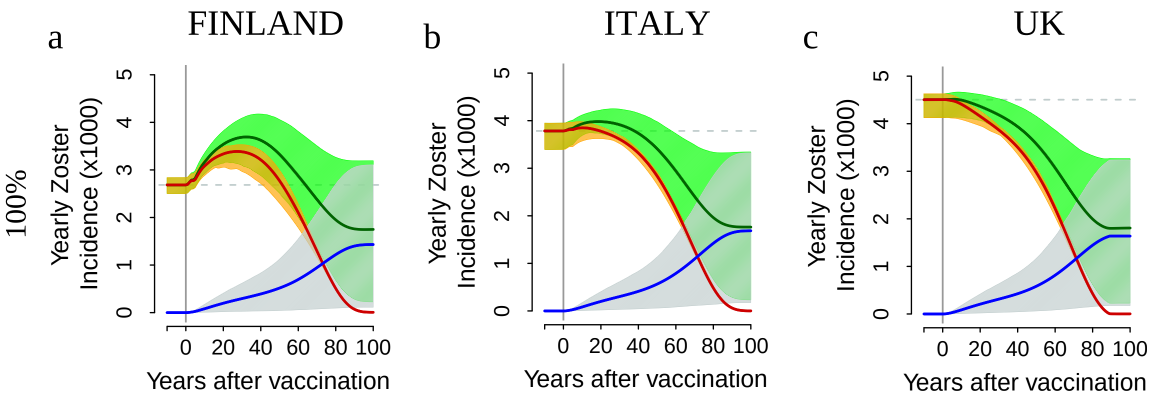

Figure S12: **Sensitivity analysis on vaccination.** Yearly incidence of HZ (average in dark green, 95% CI in light green), of natural HZ i.e., by HZ cases occurring among non-vaccinated individuals that have experienced natural varicella (average in red, 95% CI in orange) and of HZ caused by the vaccine strain (average in blue, 95% CI in light blue) per 1,000 individuals as predicted for Finland (a), Italy (b) and the UK (c) by simulating a single dose administered to 1 year old infants with 100% of coverage and by considering a more uncertain scenario where $T \in (0.7, 1)$ and $\chi \in (0.1, 0.8)$.
